# Lipid Droplet Formation, Their Localization and Dynamics during *Leishmania major* Macrophage Infection

**DOI:** 10.1371/journal.pone.0148640

**Published:** 2016-02-12

**Authors:** Sameh Rabhi, Imen Rabhi, Bernadette Trentin, David Piquemal, Béatrice Regnault, Sophie Goyard, Thierry Lang, Albert Descoteaux, Jost Enninga, Lamia Guizani-Tabbane

**Affiliations:** 1 Institut Pasteur de Tunis, Laboratoire de Parasitologies médicales biotechnologies et Biomolecules, University of Tunis El Manar, 13, Place Pasteur – B. P. 74, 1002, Tunis-Belvedere, Tunisia; 2 Université de carthage, Sidi Bou Said, Avenue de la République – B. P .77. 1054, Carthage, Tunisia; 3 Biotechnology and Bio-Geo Resources Valorization Laboratory (LR11ES31); Higher Institute for Biotechnology - University of Manouba, Biotechpole of Sidi Thabet, 2020, Sidi Thabet, Ariana, Tunisia; 4 Acobiom Cap Delta-Biopôle Euromédecine II. 1682, rue de la Valsière – 34184, Montpellier, Cedex 4, France; 5 DNA Chip Platform, Genopole, Institut Pasteur de Paris, 25–28 rue du Dr Roux, 75015, Paris, France; 6 Institut Pasteur, Département Infection et Epidémiologie, Laboratoire des Processus infectieux à Trypanosomatidés, 26 rue du Dr Roux, 75724, Paris, Cedex 15, France; 7 INRS-Institut Armand Frappier and Centre for Host-Parasite Interactions, 531, boulevard des Prairies, Laval (Québec), H7V 1B7, Canada; 8 Institut Pasteur, Dynamics of host-pathogen interactions Unit, 25 Rue du Dr. Roux, 75724, Paris, France; National Center for Cell Science, INDIA

## Abstract

*Leishmania*, the causative agent of vector-borne diseases, known as leishmaniases, is an obligate intracellular parasite within mammalian hosts. The outcome of infection depends largely on the activation status of macrophages, the first line of mammalian defense and the major target cells for parasite replication. Understanding the strategies developed by the parasite to circumvent macrophage defense mechanisms and to survive within those cells help defining novel therapeutic approaches for leishmaniasis. We previously showed the formation of lipid droplets (LDs) in *L*. *major* infected macrophages. Here, we provide novel insights on the origin of the formed LDs by determining their cellular distribution and to what extent these high-energy sources are directed to the proximity of *Leishmania* parasites. We show that the ability of *L*. *major* to trigger macrophage LD accumulation is independent of parasite viability and uptake and can also be observed in non-infected cells through paracrine stimuli suggesting that LD formation is from cellular origin. The accumulation of LDs is demonstrated using confocal microscopy and live-cell imagin in parasite-free cytoplasmic region of the host cell, but also promptly recruited to the proximity of *Leishmania* parasites. Indeed LDs are observed inside parasitophorous vacuole and in parasite cytoplasm suggesting that *Leishmania* parasites besides producing their own LDs, may take advantage of these high energy sources. Otherwise, these LDs may help cells defending against parasitic infection. These metabolic changes, rising as common features during the last years, occur in host cells infected by a large number of pathogens and seem to play an important role in pathogenesis. Understanding how *Leishmania* parasites and different pathogens exploit this LD accumulation will help us define the common mechanism used by these different pathogens to manipulate and/or take advantage of this high-energy source.

## Introduction

Leishmaniasis is an infectious disease caused by different species of the obligate intracellular parasite *Leishmania*. It is an important cause of morbidity in developing countries with 1.3 million new cases and approximately 20 000 deaths each year [1].

*Leishmania* has evolved multiple strategies to invade and exploit many cells for its survival, including macrophages, the major target cells for parasite replication. These cells are in the first line of defense against pathogens and play a key role in the recruitment of other innate inflammatory cells. The outcome of infection depends thus on the balance between the capacity of the parasite to circumvent the microbicidal functions of the macrophage and the ability of the cells to kill the parasite. Understanding how the parasite acts to evade the macrophage defense mechanisms and survive within these cells may help defining novel therapeutic approaches to fight leishmaniasis.

Recently, we and others have reported that *Leishmania* infection leads to alteration of different cell metabolic pathways including lipids and carbohydrates metabolism [2–4]. Particularly, we noticed the accumulation of lipid droplets (LDs; lipid bodies) in close association with the parasites nuclei in infected cells. Alterations in host cell lipid metabolism and the presence of increased numbers of lipid bodies (LBs) in host cells are emerging as a common feature in intracellular infections [5–8]. This phenomenon was described for a large variety of pathogens including viruses such as Viruses like Hepatitis C virus (HCV) [9,10] and dengue virus [11], bacteria especially *Mycobacterium tuberculosis* [12,13], *M*. *bovis* [14–16] and *M*. *leprae* [17–19], *Chlamydia pneumoniae* [20], *C*. *muridarum* [21] and *C*. *trachomatis* [22], *Salmonella typhimurium* [23] as well as for some pathogens components [24]. For parasites, LD accumulation was found to be induced by *Trypanosoma cruzi* in peritoneal macrophages either infected *in vitro* or derived from infected mice [25,26]. LD was also observed in pathological studies of organs infected by *Plasmodium berghei* [27,28] and in fibroblasts and both peritoneal macrophages and dendritic leucocytes infected *in vitro* by respectively *Toxoplasma gondii* [29] and *Leishmania amazonensis* [2,30].

Lipid droplets are cytoplasmic organelles composed of an hydrophobic core of neutral lipids (triglycerides “TG” and cholesterol esters “CE”) surrounded by a phospholipid monolayer and a growing list of associated proteins [31–34]. They exhibit important roles in regulating storage and turnover of lipids in different cells. During infection and inflammation, LD are altered potentially to protect the host against the harmful effects of different stimuli [7,35]. Pertinent to LD function in inflammation, LDs are described in various leukocyte types as rich deposits of esterified Arachidonic Acid (AA) which serve as precursors for eicosanoids synthesis. Enzymes necessary for this synthesis, including cyclooxygenases (COX) and prostaglandin E_2_ synthase (PGE_2_) have been localized within LDs [5]. Indeed, PGE_2_ generation and accumulation were positively correlated to LDs in macrophages infected with different pathogens like *Trypanosoma cruzi* [25] and BCG [14]. In addition to eicosanoids synthesis, LDs seem to be the site of cytokine, chemokine and growth factors localization [5] which may contribute to the mechanisms evolved by intracellular pathogens to survive within host cells. Taken together, this clearly shows that lipids play a key role in host defense.

On the other hand, interactions of these LDs with pathogen-containing phagosomes may offer the pathogen a high energy source with a strong impact on the survival of the micro-organisms infecting the hosts [8,36]. This is particularly relevant for *Leishmania* as this parasite has been described to exhibit incomplete *de novo* lipid synthesis and therefore must scavenge lipids from the host environment [37].

We have recently shown the formation of LDs in *Leishmania* infected BMMs [3] whereas others have shown that LDs are mainly restricted to *Leishmania* parasites [38]. Determining the origin of the LD accumulated in response to *L*. *major* infection may be thus of great interest as it may offer the opportunity to tip the balance of lipid droplets formation in favor of the host or the pathogen.

## Materials and Methods

### Ethics Statement

All mouse work was done according to the directive 86/609/EEC of the European parliament and of the council on the protection of animals used for scientific purposes. Approval for *mice* experiments was obtained from the ethic committee of Institute Pasteur of Tunis with ethics approval number 1204.

### Parasites

Promastigotes of the *L*. *major* tunisian strain GLC94 (MHOM/TN/95/GLC94 zymodeme MON25) were grown at 26°C in RPMI 1640, supplemented with 10% heat inactivated foetal calf serum, 2mM L-glutamine, penicillin (100 U/ml) and streptomycin (100 μg/ml). Metacyclic parasites obtained through a Ficoll gradient [3], were used in transcriptomic analysis experiments and in the first part of the Lipid droplet investigation using confocal microscopy. All other experiments were done using stationary phase cultures of *Leishmania* as the results obtained with the Ficoll gradient purified parasites were similar to those obtained with stationary phase promastigotes.

### Cell isolation and culture

BALB/c mice were killed and hind legs removed for bone marrow derived macrophages (BMMs) isolation. Briefly, femurs and tibias were flushed with HBSS (Gibco) using a 25-gauge needle. Contaminating erythrocytes were lysed through the addition of NH_4_Cl (0,17 M pH 7,35). All cells were incubated in T75 culture flasks at 1.5 x 10^6^ cell per ml in complete media additionned with 80ng/ml M-CSF (Peprotech, Neuilly sur Seine, France) overnight for stromal cell elimination. Non-adherent, immature macrophages were transferred to fresh culture-treated Petri dishes (Nunc, USA) and grown for 7 days, with re-feeding on day 3, to induce macrophage differentiation. Generated macrophages were assessed by flow cytometry for expression of F4/80 (around 90% were positive). Cells were then cultured in complete medium without M-CSF. We made sure that until 96 hours after infection, the BMMS grown in complete medium were not undergoing apoptosis.

### Cell infection

BMMs were incubated at a parasite to cell ratio of approximately 10:1 with Ficoll purified metacyclic or stationary phase promastigotes of *L*. *major*. After the desired time of incubation at 37°C in 5% CO_2_, non-ingested parasites were removed by several washing. Similar treatment was applied to the control cells. Standard Giemsa staining was used to determine the percentage of infected cells and to insure for homogenous cell infection under the different conditions. Cells were harvested in Trizol reagent to prepare samples for microarray analysis and quantitative real time PCR. For microscopy experiments, cells were either fixed for slide preparation or directly stained for live cell imaging.

### RNA isolation, microarray hybridization, analysis and normalization

One million macrophages were lysed directly in 1ml Trizol reagent (Invitrogen). Total RNA from uninfected and infected macrophages were prepared using the RNeasy mini kit (Qiagen) and treated with DNAse according to the manufacturer’s protocol. 100 ng of RNA from each biological condition were amplified and labeled with biotin according to the GeneChip whole transcript sense target labeling assay manual and using the GeneChip WT cDNA Synthesis and amplification Kit and WT terminal labeling Kit. The fragmented and labeled ssDNA was hybridized to the GeneChip Mouse Gene 1.0 ST array (Affymetrix, Santa Clara, CA), washed with the Fluidics station 450 and scanned using the Affymetrix Scanner 30007G. Each infection and control time points were performed on three different samples, using different preparations of BMMs, and processed independently to give three biological replicates.

Expression analysis were performed using the Limma package to identify genes that met statistical significance (*P* < 0.05 after adjustment according to the method of Benjamini and Hochberg and fold-change criteria (at least a 1.5-fold change) for differential expression using the following contrasts: macrophages infected with live parasites at a given time point versus non infected macrophages incubated with vehicle (media) for the same time. The same contrast was used for heat-killed *Leishmania*-infected macrophages. Macrophage genes modulated during the kinetics were first detected.

In accordance with MIAME (Minimum Information About a Microarray Experiment) regulations, all data were deposited into GEO (Gene Expression Omnibus) database at www.ncbi.nlm.nih.gov/geo/ under the accession number GSE31995.

### dchip clustering

The affymetrix 1.07 array data related to lipid pathways were analysed using dChip software (http://www.biostat.harvard.edu/~cli/dchip_2010_01.exe) in order to identify clusters regulated later or earlier throughout the kinetic of *Leishmania*. *major* infection between Live and Killed parasite infection.

### Ingenuity global functional analysis

Ingenuity Pathways Analysis (IPA; Ingenuity, CA; Systems 2008) tool was used to identify relevant lipid canonical pathways in macrophages that were altered by *Leishmania* parasite infection, based on a Fischer’s exact test to calculate a p-value for each biological function founded (at least *P* < 0.01). A list of the statistically significant differential genes expression in *L*. *major*-infected BALB/c macrophage related to lipids for each measured time point was generated and mapped to their functional networks in the IPA database. This analysis shows the number of *Leishmania* modulated genes belonging to a given pathways which are ranked by the obtained score. The relationships between the generated networks and known pathways were then investigated using the canonical pathway analysis function.

### Quantitative real time PCR

RNA quantity, was controlled using NanoDrop ND-1000 micro-spectrophotometer and RNA quality and integrity (RNA Integrity Number, RIN>9) was monitored on Agilent RNA Pico LabChips (Agilent Technologies, Palo Alto, CA). Reverse transcriptions were performed for each of the selected mice samples in 20μl final reaction volume with 273 ng of total RNA using 200 Units of SuperScript III enzyme (M-MLV RT, Invitrogen) and 250 ng of random primers according to manufacturer's instructions (25°C 10 min, 42°C 50 min, 70°C 15 min). All RT reactions were performed the same day with same pipette set and same manipulator. A negative control was included by performing a RT with no template. qPCR experiments were carried out using EVA Green chemistry on a microfluidic multiplex qPCR apparatus (BioMark, Fluidigm) following manufacturer's instructions. For each cDNA sample, a Specific Target Amplification (STA) was performed with a pool of primers targeting all selected genes (Pre-Amplification of 14 cycles using TaqMan PreAmp Master Mix (Applied Biosystems) and following manufacturer's instructions): Each qPCR was performed with 1/20 STA dilution, in duplicate. Relative gene expression kinetics was created by a first normalization with 4 reference genes followed by a second normalization with Non Infected macrophage cells (NI). Values are expressed in fold changes (2-Delta Delta Ct Method) compared to NI macrophage cells.

### LD evaluation in fixed cells

Twenty four hours post infection, macrophages were fixed in 4% paraformaldhehyde for 10 min at room temperature then washed 3 times in 1X PBS. For LD staining, Bodipy 493/503 dye (4,4-difluoro-1,3,5,7,8-pentamethyl-4-bora-3a,4a-diaza-s-indacene, Molecular Probes) was used as previously described [3]. Cells were permeabilized using 0,1% TritonX-100 and non-specific surface binding was blocked in blocking buffer (1% bovine serum albumin (BSA), 20% goat serum, 6% milk and 50% FBS) for 20 min at room temperature. To visualize the LD/parasitophorous vacuoles interaction, cells were then incubated with anti-LAMP1 (1D4B) primary and to better show LD localization in intracellular parasites, cells were incubated with mouse monoclonal antibody against LPG (CA7AE) from Cedarlane Laboratories OVN at +4°C both, followed by Alexa Fluor 568-conjugated anti-Rat IgG secondary antibody (Molecular Probes). Cell and parasite nuclei were stained with DRAQ5 (Biostatus, Leicestershire, UK). To evaluate the role of endocytosis in LD formation, cells were incubated in the presence of red fluorescent latex beads (50/1; Polysciences) for 24 hours and then stained with Bodipy.

All coverslips were mounted on slides with Fluoromount-G (Southern Biotechnology Associates). Preparations were examined using a Zeiss LSM780 confocal microscope equipped with 30 mW 405 nm diode laser, 25 mW 458/488/514 argon multiline laser, 20 mW DPSS 561 nm laser and 5 mW HeNe 633 nm laser mounted on Zeiss Axio Observer Z1 and operated with Zen 2011 software (Zeiss). We used a Plan-APOCHROMAT 63× oil DIC 1.4NA objective for our observations and images were acquired via sequential acquisition. The images were edited using Zen bleu (Zeiss) and ImageJ 1.47 (National institute of Health, USA). The morphology of the fixed cells was observed, and LDs or LD positive cells were enumerated at 63X in 100 consecutively scanned cells for at least 4 independent experiments.

### Generation of DsRed- transgenic *L*. *major* GLC94 parasites

The 697-bp DsRed2 coding region was cut by *Bam*HI/*Not*I from pDsRed2 (Clontech, CA) and subsequently cloned into *Leishmania* expression vector pF4X1.HYG (Jenabioscience, Jena, Germany) previously cut by *Bgl*II/*Not*I. This plasmid contains the Hygromycin B (Hyg) marker for the selection of transgenic *Leishmania*. In this construct, the 3’ and 5’UTR regions flanking the DsRed2 and HYG genes provide the required splicing and polyadenylation signals. Following linearization with *Swa*I, transfections were performed by electroporation using the Nucleofactor technology (Amaxa-Lonza). Cells were incubated for 24 h in normal medium, and then with Hygromycin B for 96 h in liquid medium. They were finally, plated for selection of transgenic *Leishmania* on semi-solid medium containing 50 μg/ml of hygromycin B (Cayla, Toulouse, France). Recombination of the engineered plasmids leads to integration into the *Leishmania* rDNA locus, ensuring a permanent high level of transcription.

### LD evaluation in live cells

For live imaging, macrophages were seeded in 96 well plates (Optical Bottom Plate, Black Polystyrene, Cell Culture Treated and Sterile; Thermo scientific) and infected with transgenic Ds-Red or WT parasites for time-courses ranging from 15 min to 24 h. Extracellular parasites were then washed out and cells were stained with Bodipy for LD tracking. 3D confocal time-lapse imaging was performed at 37°C using a PerkinElmer UltraVIEW VoX spinning disk attached to an Eclipse Ti inverted microscope (Nikon) with a 40X/1.3 N.A. dry objective. Images were collected sequentially in two channels (488 and 561nm laser) every 5 to 30 min for 1, 3, 6, 12, 18 and 20 h post infection. Acquisition was performed and analyzed using Volocity analysis software. Quantification of LD accumulation in contact with *L*. *major* parasites during the time lapse experiment was performed using ImageJ software. We measured green fluorescence intensity (488nm) in a same region of interest (ROI) corresponding to a DsRed-parasite localized in cell cytoplasm, through the Measure plugging analysis.

### Statistical analysis

Results are representative of at least 3–6 independent experiments. The data are presented as the mean plus SEM (standard error of the mean) and were analyzed using the Graph- Pad Prism 5.03 software (GraphPad Software, San Diego California USA, “www.graphpad.com”). The experiments were analyzed using One-way analysis of variance (ANOVA) with Dunnett’s or Bonferroni’s test for the comparaison of three or more groups of data and Unpaired Student T-test for the comparaison of two groups of data. The differences were considered statistically significant when p <0.05 (*), p <0.01(**) and p <0.001 (***).

## Results

### Bioinformatic analysis of transcriptomic data related to lipid pathways

GeneChip Mouse Gene 1.0 ST arrays were used to analyse global changes in gene transcripts induced by *L*. *major* infection of bone marrow derived macrophages, and generated a pool of genes that was statistically significant (p-value < 0.05) with a fold change cut-off of 2. As previously reported in our transcriptomic analysis, canonical metabolic pathways including carbohydrate and lipid metabolisms are among the most altered pathways [3]. According to the importance of lipids in infection, we decided to focus on studying the modulation of lipid pathways related genes upon *Leishmania* infection.

**Fig 1:**
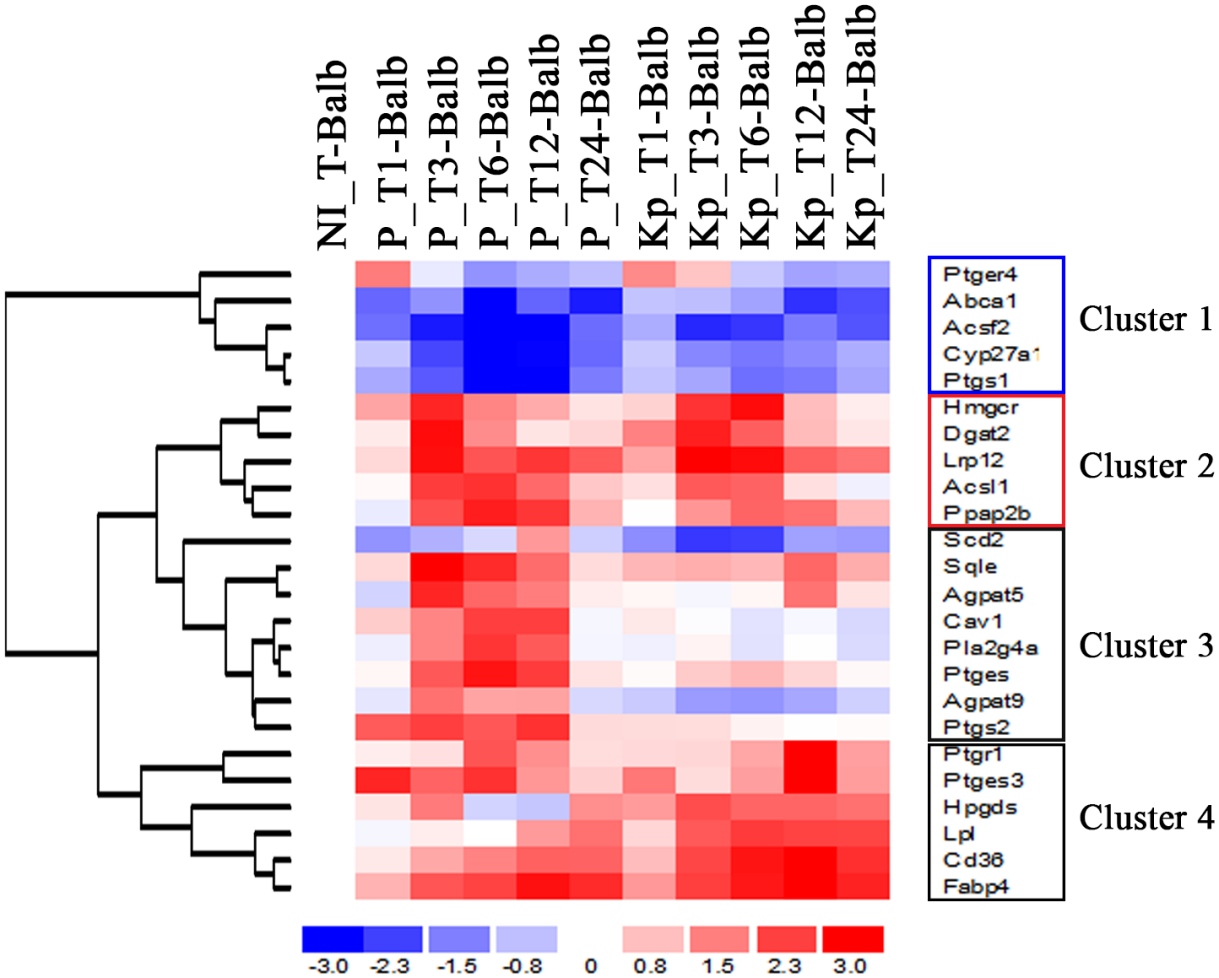
Hierarchical clustering of lipid metabolism related genes that are differentially expressed in *L*. *major* infected BALB/c derived macrophages. Analysis using dChip software of lipid related transcripts identified four distinct clusters. Cluster 1 contains genes down-regulated in both live parasites (P) and heat-killed parasites (KP)-infected cells. The cluster 3 contains genes up-regulated in P and down-regulated in KP-infected cells. Cluster 4 contains genes more heavily up-regulated in KP- infected cells. Finally, cluster 2, contains genes up-regulated during early infection times (3 and 6 h pi) in both P and KP. Each row of the cluster represents a spot on the microarray and each column a separate microarray. The BMM response was studied at five different time points (from the left to the right: T1h, T3h, T6h, T12h and T24h) in P and KP-infected cells. The left-hand column shows non-infected cells (NI) that were used as internal control.

Comparing to the control (non-infected), the lipid related genes modulated by live (P) and killed parasites (KP) revealed their differential clustering upon infection (Fig 1). The first cluster contains the entire set of genes down-regulated by infection with a more important signal shown in cells infected by live parasites at late time points, while Cluster 2 includes genes up-regulated during the first hours of infection by both P and KP. Cluster 3 includes genes up-regulated exclusively by live parasites from 3 to 24h post infection which includes in particular, PLA2, Ptgs2 (COX-2) and Ptges while cluster 4 contains genes which transcription is more heavily up-regulated by KP across the kinetic of infection. In another hand, some genes (Agpat, Dgat2 and Pap2b, all key enzymes in triacylglycerol biosynthesis) belonging to the specific biological pathway of lipid droplet formation are distributed among different clusters and are not strictly activated by live or heat killed parasites.

**Fig 2:**
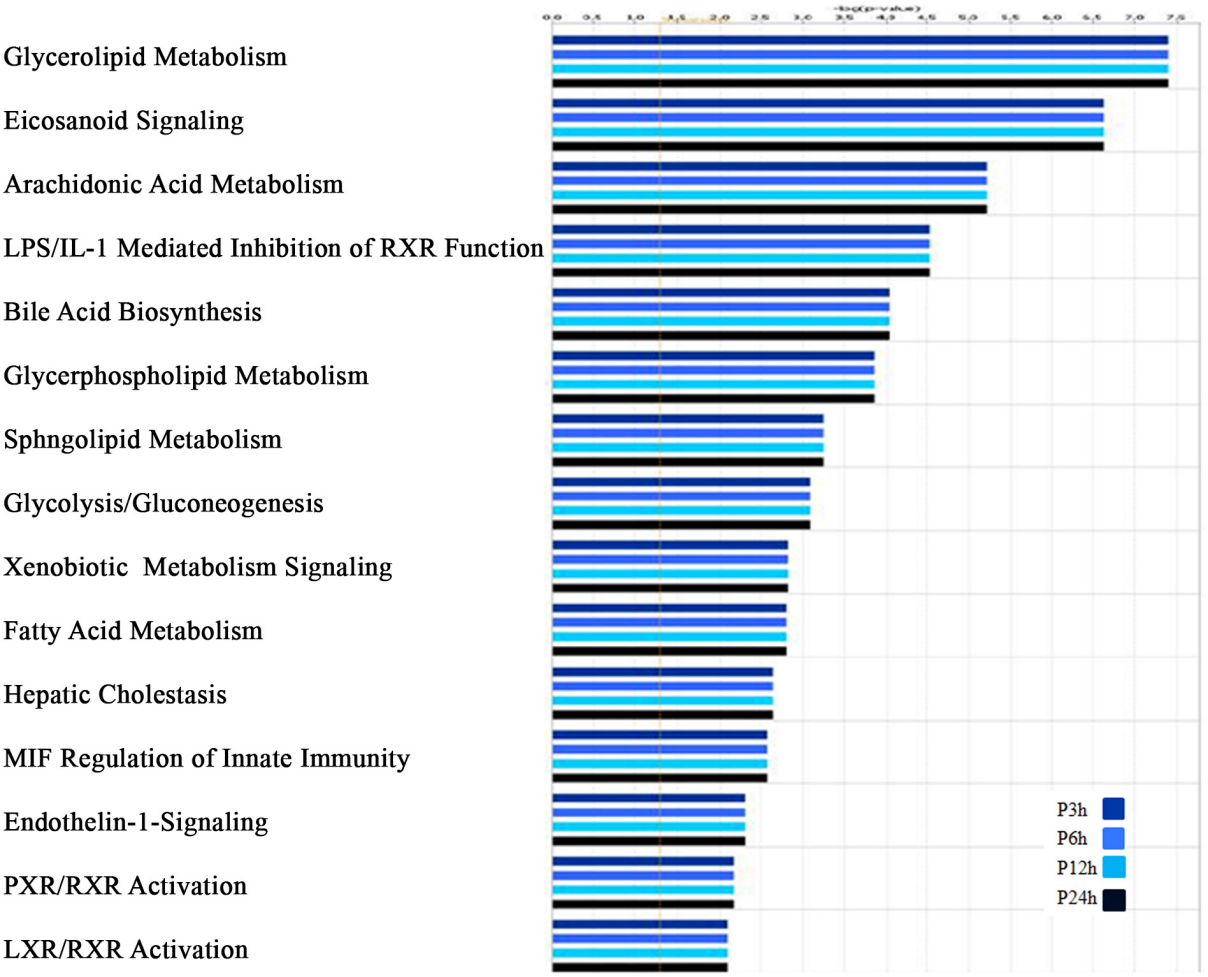
IPA analysis of Lipid related genes. Lipid related metabolic pathways identified by Inguenuity Pathway Analysis (IPA) software as significantly altered (p < 0.05) in *L*. *major* infected BALB/c macrophages for all time points. Each bar graph shows the number of *Leishmania* modulated genes belonging to a given pathways and ranked by their scores. The negative log_10_(p-value) is plotted on the Y-axis.

Furthermore, the set of genes related to lipid pathways was also analysed through Ingenuity Pathways Analysis Software (Ingenuity Systems, Redwood City, CA; http://www.ingenuity.com) (IPA). Even if these genes are expressed with different dynamics throughout the kinetic, the final number of modulated genes remains the same for each time point which explains the absence of variation of each lipid pathway across the time course of infection. Nevertheless, this analysis reveals according to their score that glycerolipid, eicosanoid and fatty acid metabolisms are among the top ten lipid pathways modulated by the parasite (Fig 2 and S1 Table). In addition, a number of genes coding for lipid droplets related proteins involved in the de novo TAG biosynthesis and hydrolysis and the accumulation of cholesterol are found significantly activated in *L*. *major* infected BMMs (S2 Table, S1 and S2 Figs).

### Time course of LD accumulation in *L*. *major* infected macrophages

**Fig 3:**
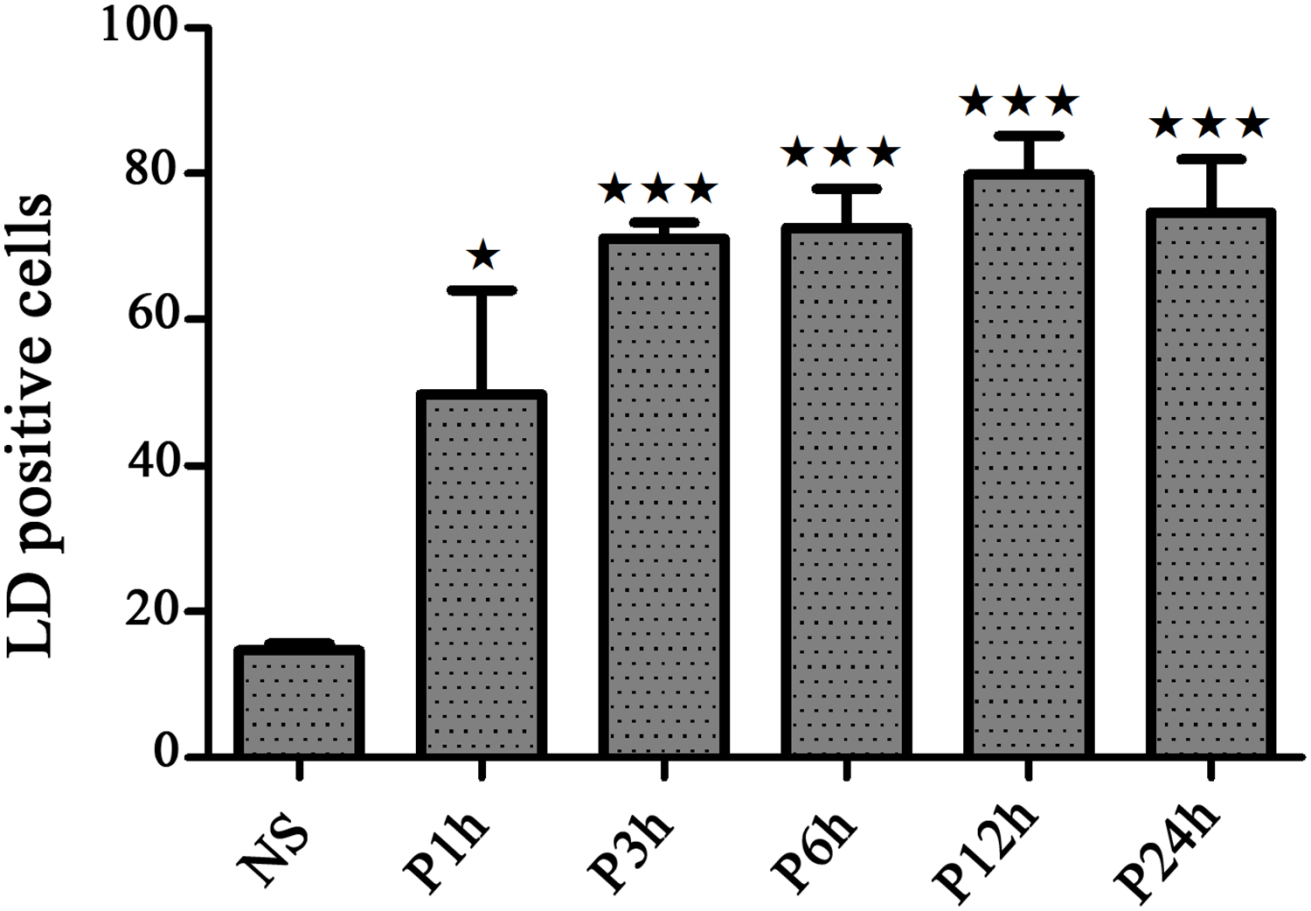
Time-course of LD accumulation in *L*. *major* infected BMMs. BMMs were infected for different times with live parasites. Cells were then fixed and subjected to Bodipy 493/503 staining for lipid droplets accumulation enumeration. Each bar represents the mean plus standard error of the mean (SEM) from 100 consecutively counted macrophages from at least 4 independent experiments. Statistically significant (*, p <0.05) and (***, p <0.001) differences between control and infected groups are indicated by asterisks.

The formation of LDs is closely linked to the biosynthesis of triglycerides and cholesterol esters, hence we investigated the role of *Leishmania* infection on LD formation in infected BMMs. Cells were infected with *L*. *major* promastigotes and the presence of LDs was examined at various time points post-infection by immunofluorescence microscopy using Bodipy 493/503. Analyzing the time course of LD formation after infection, we show (Fig 3 and S3 Fig) that compared to non-infected cells, the number of *Leishmania*-infected cells displaying LD accumulation is significantly enhanced. This increase is time dependent, peaked at 3h post-infection and remains significantly heightened. Similar results were observed in cells infected with heat-killed promastigotes.

### Prostaglandins status in *L*. *major* infected BMMs

LDs were shown to play central roles in the heightened production of eicosanoids, thus participating in the amplification of the inflammatory response and ultimately regulating the host immune response to intracellular pathogens. We thus investigated whether the accumulation of LDs in *L*. *major* infected cells correlates with PG production.

Our transcriptomic data show that among the set of genes exclusively up-regulated by live parasites (Fig 1 cluster 3), we found in particular those coding for PLA2 that catalyzes the hydrolysis of plasma membrane phospholipids to lysophospholipid and arachidonic acid the substrate of Ptgs2 (also known as COX2) and Ptges (prostaglandin E synthase) that catalyzes the production of prostaglandin E2. The data from RT-qPCR analysis (Table 1) is consistent with the results obtained by microarrays, albeit with magnitudes different from, and often higher than, those recorded by the microarray analysis.

**Table 1:** Prostaglandin modulated genes in *L*. *major* infected BMMs validated by qPCR. Gene expression of prostaglandin pathway related genes in BALB/c *Leishmania* infected macrophages using qRT-PCR is analysed. Changes in mRNA levels are calculated using the 2^−ΔΔC*T*^ method (^ΔΔ^Ct = ^Δ^Ct -^Δ^Ct NI (t: point of the infection with P or KP) and ^Δ^Ct = Ct gene of interest- Ct of reference gene). Four reference genes were used for the qPCR normalization. The numbers presented for each time points are the average of the three biological replicates. The abbreviations NI, P and KP respectively refer to Non Infected macrophages, macrophages infected with live Parasite, macrophages infected with Killed Parasite.

MGI Symbol	P1h	P3h	P6h	P12h	P24h	KP1h	KP3h	KP6h	KP12h	KP24h
**Pla2g4a**	1.0801553	2.3360319	4.1006438	1.5113453	1.2962157	1.2332394	1.5805938	1.7544114	0.9620814	1.1038153
**Ltb4dh**	0.917572	1.4579723	3.7112559	1.7832729	1.565753	0.9108592	1.4451228	3.5579161	1.9362804	1.2644033
**Ptgds2**	0.8647273	1.5389983	0.640017	0.4710319	0.8149598	0.8600334	1.7178928	0.8497618	1.0564791	1.0370536
**Ptger4**	2.2779805	1.1879741	0.7858262	1.0733496	1.0209207	2.1385994	2.5182154	0.8230482	1.0560935	0.9784231
**Ptges**	1.4022955	3.9840341	17.936259	3.635398	2.7507897	1.9853264	1.8932392	4.0471007	1.5335914	1.4483317
**Ptges3**	1.0452493	1.3586009	1.3767059	0.7188943	0.8588854	1.0893767	1.3420654	1.2157013	1.1292468	0.7800727
**Ptgs1**	0.9768465	0.3645247	0.1209849	0.3099506	0.4500792	0.9234279	0.4723992	0.1755423	0.5141908	0.7198303
**Ptgs2**	8.3963778	5.3499114	10.167681	4.9344011	1.8940387	3.2175285	4.7326995	2.033005	0.9532793	0.8005539

### Multiple factors are involved in LD accumulation within *L*. *major* infected macrophages

**Fig 4:**
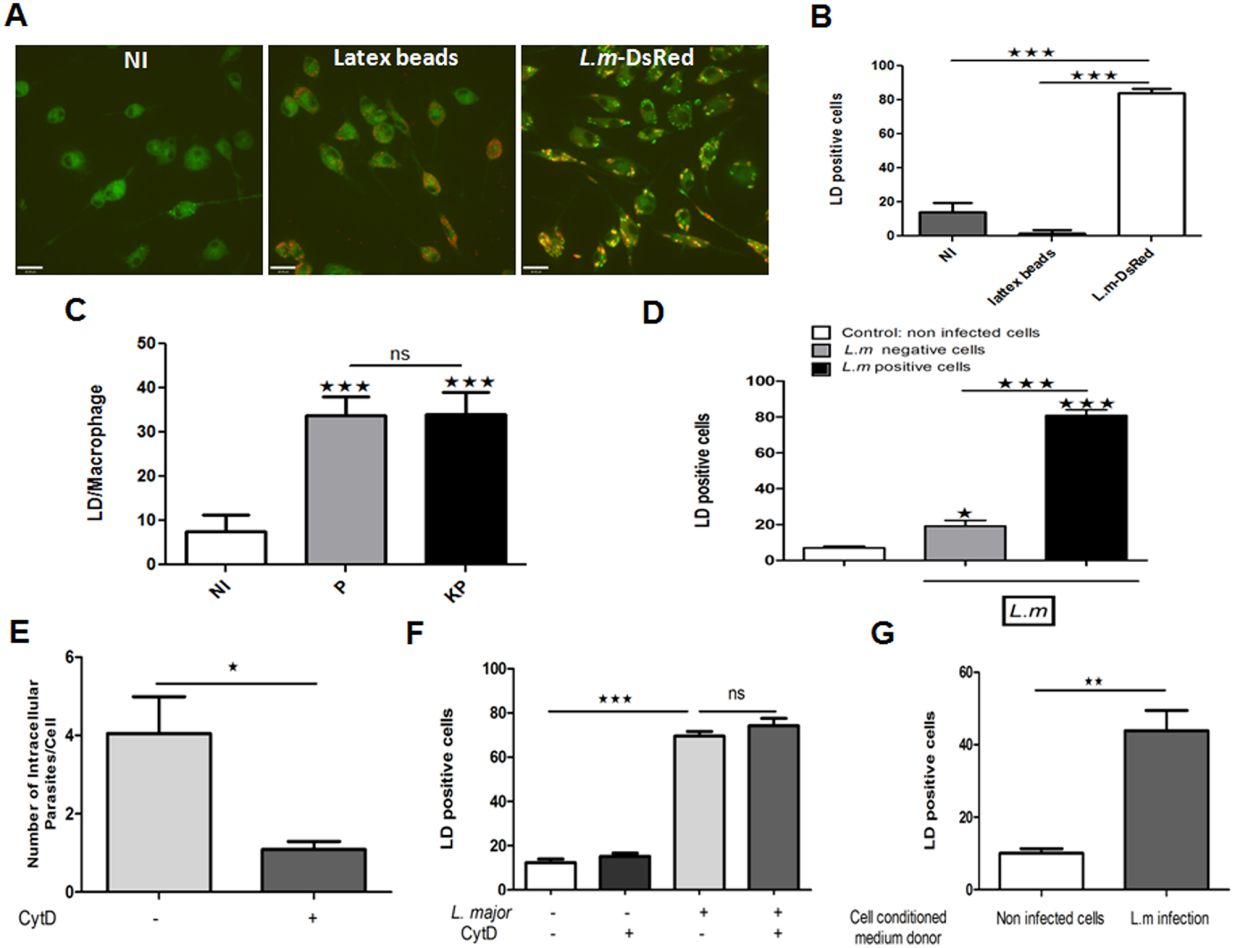
*L*. *major* induced LD formation in BMMs infected with live and killed parasites and in non-infected neighbouring cells (A) LDs (green) were visualized after Bodipy staining, 24h post treatment with DsRed-*L*. *m* (red) or fluorescent latex beads (red). (B) Quantification of LD in red latex beads stimulated and *L*. *major* infected BMMs, by enumeration of LD positive cells after Bodipy 493/503 staining. (C) Enumeration of LDs after Bodipy staining in BMMs infected by live and heat-killed parasites. (D) Bar graphs show LD positive cells in *L*. *major* containing and *L*. *major* free cells. (E) Quantification of cytochalasin D effect on *L*. *major* internalization in BMMs. (F) Lipid droplets positive cells were quantified in non-infected and *Leishmania* infected macrophages previously treated or not with cytochalasin D. (G) Quantification of LD positive cells was performed in naïve cells exposed for 24 hours to non infected or *L*. *major* infected cells conditioned medium. Statistically significant (*, p <0.05), (**, p <0.01) and (***, p <0.001) differences between control and infected groups are indicated by asterisks. Data are representative of four independent experiments and are expressed as the mean plus standard error of the mean (SEM).

To determine whether the accumulation of LDs in *L*. *major* infected macrophages is a process actively regulated by the parasite, we incubated murine BMMs either with latex beads, live or heat-killed promastigotes. Twenty four hours post-infection, the presence of LD was examined by immunofluorescence microscopy using Bodipy 493/503. Fig 4A, 4B and 4C show that live and heat-killed promastigotes known to be phagocytosed by macrophages [39] but not latex beads, induced a similar and significant accumulation of LDs (Fig 4C) suggesting that accumulation of LDs is probably not triggered by phagocytosis *per se* and that the stimulation of the receptors involved in the recognition of *Leishmania* parasites is sufficient to induce such process. To test whether phagocytosis is required to allow LDs formation, cells were treated with cytochalasin D, an inhibitor of actin polymerization, for one hour and its effect monitored by confocal microscopy upon addition of wild type as well as Ds-Red *Leishmania* parasites. Fig 4E and 4F, show that while this treatment significantly inhibited parasites internalization it has no effect on lipid droplets formation.

On the other hand, LD formation described herein was not restricted to cells bearing parasites. Indeed, our result presented in Fig 4D shows that LDs are present in macrophages containing however, significantly higher LD formation in *L*. *major* bearing cells.

This result suggests that soluble factors produced by parasites infected cells might contribute to lipid accumulation in non-infected macrophages. We thus investigated the ability of *Leishmania*-infected cells supernatant (*L*. *m* conditioned medium) to elicit LDs formation. Supernatant obtained from macrophages infected for 24 hours or not were used to treat naïve cells for 24 hours. Fig 4G; show that *L*. *major* conditioned medium is able to elicit a significant LDs formation relatively to non-infected cells conditioned medium, confirming that soluble factors secreted from infected cells are able to induce LDs formation in free parasites cells.

### Localization of LD in *Leishmania major* infected macrophages

To study the dynamics of the LD–*Leishmania* association in living cells, a time-lapse sequence of BMMs infected with DsRed transgenic parasites was investigated using confocal microscopy.

**Fig 5:**
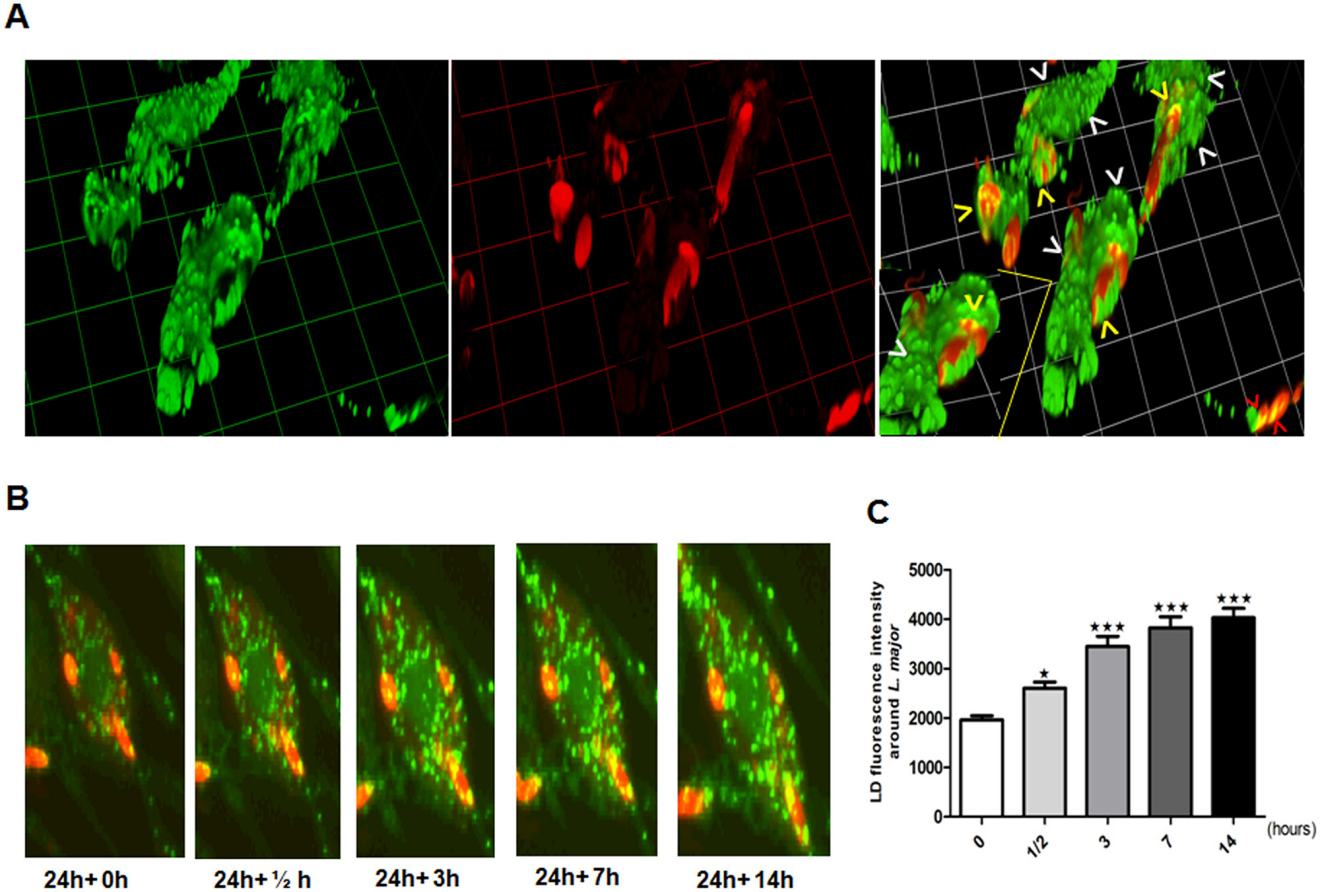
LD accumulation in live Ds-red-*L*. *major* infected BMMs. Macrophages were infected with the transgenic fluorescent parasites Ds-Red-*L*. *major* GLC94. The LDs in Lm-infected BMMs were stained with Bodipy and time-lapse sequence of the recruitment processes was investigated using live time confocal microscopy. (A) 3D images were acquired by confocal microscopy through the time-series of Z-stacks and 3D representation was performed using the opacity tool (Volocity). LDs presence was detected in the cell cytoplasm (white arrowheads), in internalized parasites (yellow arrowheads) as well as in extracellular parasites (red arrowheads). (B) Representative time-lapse series examining late-time course of LD recruitment during *L*. *major* infection. Neutral lipids in the cytoplasm (LDs) were stained with Bodipy after 24 h of parasitic infection and time-lapse photography of the cells at 30 min intervals for 18 hours was done. Images clearly show that parasites (red) are massively surrounded by LDs (green) across the acquisition time. (C) Quantification of LD accumulation coming in contact with *L*. *major* parasites during the time lapse series presented in B, was performed using ImageJ software. Statistically significant (*, p <0.05), and (***, p <0.001) differences between control (point 24h+0h) and the four different time points are indicated by asterisks. Data are representative of three independent experiments and are expressed as the mean plus standard error of the mean (SEM).

**Fig 6:**
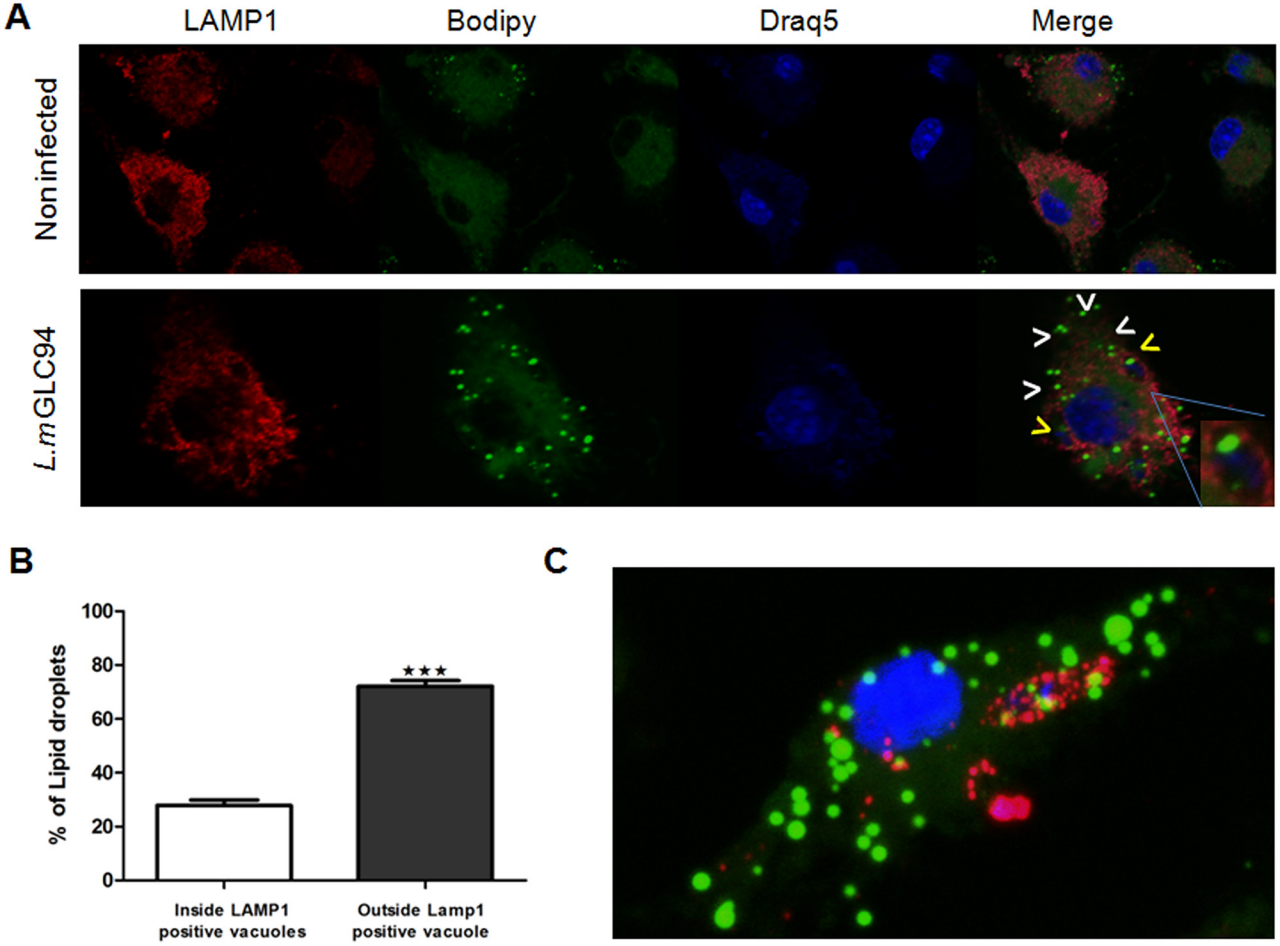
LD formation is neither restricted to parasitophorous vacuoles containing *L*. *major* nor to LPG stained *Leishmania* parasites in infected BMMs. BMMs were infected with WT *L*. *major* parasites for 24h. Cells were then washed to remove extracellular parasites. (A) Parasitophorous vacuoles were stained using anti-LAMP1 and nuclei were stained using Draq5. Cells were then incubated with Bodipy for Lipid Droplet localization. White arrowheads show LD out of the LAMP1 stained vacuoles while yellow arrowheads show LD localized inside the parasitophorous vacuoles. Lipid accumulation inside the parasitophorous vacuole is shown in a magnification box. (B) Quantification of lipid droplets percentage inside and outside the LAMP1 positive parasitophorous vacuole in Leishmania infected macrophages. Statistically significant (***, p <0.001) difference between the percentage of LD inside and those outside the LAMP1 stained vacuole are indicated by asterisks. Data are representative of three independent experiments and are expressed as the mean plus standard error of the mean (SEM). (C) LD localization in cell cytoplasm as well as in parasite cytoplasm is shown by a Z-stacks and 3D image rendering. Cells were subjected to anti-LPG for parasite outline, Bodipy for LD and Draq5 for nuclei staining. Different snapshots of LD localization around and in parasite cytoplasm from the 3D image rendering were pinpointed in magnification boxes.

The accumulation of LDs appears as early as 15 min post infection and massive LD accumulation is observed throughout the measured time-course. Our results also show, that while most of the LDs are found in parasite free cytoplasm, a subset of LDs co-localizes with the intracellular DsRed parasites. Interestingly, we also notice using live cell imaging, an LD accumulation on some of the extracellular infectious parasites as observed by 3D image reconstruction (Fig 5A). We thus quantified LD recruitment from cell cytoplasm to intracellular parasites during the live time lapse imaging by measuring LD fluorescence intensity coming to the contact of DsRed-parasites using ImageJ software. Our result shows a significant direct cumulative recruitment of LDs to the DsRed-parasites in live time-lapse (Fig 5C). However, we also show that most of the LDs are not restricted to the proximity of DsRed parasites. Moreover, the simultaneous labeling of infected cells with LAMP1 antibody and Bodipy 493/503 in fixed cells at 24h post infection also shows that the presence of LD is not restricted to the LAMP1 stained parasitophorous vacuoles (Fig 6A). The quantification of lipid droplets accumulation inside and outside the LAMP1 positive parasitophorous vacuoles, clearly, shows an accumulation of LDs inside the LAMP1 positive vacuole but also a significant accumulation outside of the parasitophorous vacuole (Fig 6B).

Finally, a 3D representation using volume-rendering, of infected cells simultaneously labeled with an antibody specific to parasite surface lipophoshphoglycans (LPG) and Bodipy 493/503 shows the presence of LD inside the parasites but also, and to a large extent, in different parasites-free cytoplasmic regions of the cell (Fig 6B and S1 Video). LDs dynamic monitoring, by a time-lapse recording was carried out starting at 30min, 1h, 3h, 6h, 18h and 24h after *Leishmania* infection with intervals of 5 to 30 minutes. A continuous LD movement occurring in the cytoplasm and around the parasites at both early and late time points is evident (Fig 5B, S2 Video and S3 Video) indicating a highly dynamic behavior of LDs in *L*. *major* infected macrophages suggesting a possible continuous interaction with the parasitophorous vacuole and *L*. *major* parasites.

## Discussion

Intracellular pathogens have evolved multiple strategies to escape the host immune system and survive within host cells. The modulation of host lipid metabolism through targeting LD formation and accumulation has been described for some of them, and emerges as a common phenotype to viral, bacterial and parasitic infections [7]. In addition, it has been recognized that LDs are involved in different aspects of inflammation [6]. However, to date the understanding of LD formation and their dynamics in the interplay between hosts and parasites is still limited.

In this study, we evaluate the effect of *L*. *major* infection on lipid related genes transcripts and on cellular LD formation and dynamics and provide novel insights into the origin of the formed LDs.

Our study shows that upon *L*. *major* infection transcriptomic analysis reveals cholesterol uptake and an activation of *de novo* triacylglycerol (TG) synthesis which together suggest the formation of LDs. The modulation of these lipid pathways has also been described in dendritic leukocytes infected with *L*. *amazonensis* but with different mechanisms [2]. Our results also offer the evidence that LD accumulation in infected macrophages is time dependent. It initiates very quickly after bringing into contact the cells with the live or heat-killed parasites and the number of LDs positives cells increases steadily between 1 and 3 h post-infection. We then, observe an increase in the size and the number of LDs per cell (data not shown). These changes could be explained by a simultaneous packaging of the newly synthesized TG and CE and an insertion of these newly synthesized neutral lipids in the hydrophobic core of existing LDs [40]. Our microarray also shows the transcriptional activation of several genes such as Diacylglycerol O-acyltransferase 2 (Dgat2) in response to *L*. *major* infection [3], described as critical for LD biogenesis. Indeed, Dgat2 have been shown to be a component of a triglyceride synthesis complex that facilitates LD expansion and that a loss of Dgat2 function blocked LD expansion [41,42]. This modulation in lipid homeostasis has been observed for several pathogens including *Staphylococcus aureus*, Epstein-Barr virus, Herpes simplex virus, HIV, *Schistosoma* and *Plasmodium* infections and pathogens components like *M*. *tuberculosis*-derived lipoarabinomannan (LAM) and *E*. *coli* LPS [35,36]. According to the role of LDs in eicosanoids synthesis and storage [7,43], our transcriptomic data show that live parasites but not killed ones, induce the transcriptional up-regulation of the genes coding for PLA2, COX2 and Ptges proteins and enzymes implicated in the synthesis of prostaglandin (Fig 1, cluster 3). Turning off macrophages' microbicidal property may be obtained by upregulation of COX-2, as well as immunosuppressive PGE-2 production. Indeed, the higher plasma levels of prostaglandin E2 (PGE2) measured in patients with DCL, compared with patients with localized cutaneous leishmaniasis (LCL) or with controls from an area of endemicity [44], and the effect of PLA2 through the generation of PGE2 in the progression of cutaneous leishmaniasis [45] underline the key role of this inflammatory lipid mediator, in the modulation of the host immune response. Interestingly, secretory serine protease (pSP) has been involved in down-regulation of macrophage microbicidal activity by inducing host inflammatory responses in terms of COX-2-mediated PGE-2 release [46], suggesting that parasite viability is crucial. Our results are consistent with these data as they show that stimulation of PLA2-COX2-PGE2 pathway that suppresses macrophage's activation is observed only in macrophages infected by live parasites. Nevertheless, as reported for *L*. *donovani* [47], we have demonstrated previously that the protein level of COX2 is unchanged following *Leishmania major* infection [3] suggesting that LDs formation may not correlate with PGE2 production in infected cells.

It has been recently shown that *Leishmania* promastigotes inhibit the macrophage translational machinery by cleaving mTOR, thereby activating the protein 4E-BP1 in order to promote its proliferation and survival into the host [48], which could influence the biologically active COX2 protein level.

We demonstrate that, in contrast to eicosanoids, LD formation is induced to the same extent by both live and heat-killed parasites suggesting that this process is independent of parasite viability and that the formed LDs should be from a cellular origin. We also show that phagocytosis of latex beads by BMMs did not trigger LD formation. Furthermore, inhibition of cytoskeleton movement and parasite phagocytosis did not abrogate LDs formation suggesting that *Leishmania* internalization in not involved in the formation of LDs and that receptors triggering and downstream signaling pathways is sufficient to induce such process. Moreover, our results show that parasite-free cells also exhibit a significant increase in LD accumulation compared to the control and that conditioned medium from *Leishmania*-infected cells is able to induce lipid droplets formation in non-infected cells. Taken together these results indicate that soluble factors in infected cells may act in a paracrine manner to induce the formation of LDs in uninfected bystander cells. Similar results have been demonstrated for different other pathogens. Indeed, *Mycobacterium bovis* bacillus Calmette Guerin (BCG), unlike latex beads and the non-pathogenic mycobacteria *M*. *smegmatis*, significantly increases cytoplasmic LD accumulation and authors suggest that the transfer of mycobarterial lipids and/or cytokines and other inflammatory mediators to uninfected bystander cells might contribute to lipid droplets formation [14]. This has also been reported for *Mycobacterium leprae* in human peripheral blood monocytes and murine macrophages [17]. In this context we show here, as observed for *T*. *cruzi* [26], that cells containing internalized pathogens, accumulate higher number of LD compared to pathogens free-cells, indicating that phagocytosis potentiates LD biogenesis.

We next monitored the localization of lipid droplets in infected cells. We either used DsRed transgenic parasites and Bodipy 493/503 labeling to perform time-lapse live cell imaging at different times post-infection or WT parasites and simultaneous LAMP1 or LPG and Bodipy 493/503 labeling of fixed cells. We demonstrate that LDs are dynamic organelles accumulating as early as 15 min post infection and are highly mobile into the cell cytoplasm. Otherwise a very fast turnover of LD is noticed since we observed a very quick recovery of Bodipy signal following fluorescence recovery after photobleaching (FRAP) (data not shown). Our results show the presence of LD in both non-infected cells and in parasites free cytoplasmic regions suggesting that there is a prominent accumulation of LDs from a cellular origin. This does not seems to be the case for *L*. *amazonensis* that were unable to trigger LD formation in dendritic cells in the absence of oleic acid [2], nor for *Leishmania infantum chagasi* that does not induce any host cell LD formation during BMMs infection and for which the LDs seem to be restricted to the parasitophorous vacuole [38]. These differences can be due to specific characteristics and adaptation strategies of the different parasites species used in all these studies.

Our results clearly show that LD also co-localize with the LAMP1 stained parasitophorous vacuoles and with the DsRed transgenic parasites or LPG stained parasites. As we also noticed an LD staining on the surface of extracellular *L*. *major* parasites some of the cytoplasmic LD, may originate from parasite exosomes trafficking outside of the parasitophorous vacuole in a phenomenon similar to the one observed for GP63 and LPG in infected macrophages [49,50]. However, using Bodipy 493/503 we were able to visualize in time lapse a precise LD recruitment to DsRed-parasites and accumulation into the LAMP1 stained parasitophorous vacuoles in fixed samples. By quantifying LD fluorescence intensity on parasites during our time lapse imaging, we confirmed the recruitment of LDs from cell cytoplasm to intracellular parasites in live cells across the time lapse experiment.

Different pathogens were also described as able to recruit LDs to their vacuoles [8]. Indeed, the triggering of macrophages by *T*. *cruzi* demonstrate a clear association of LDs with phagosomes and LD formation even within the lumen of these structures [25,51]. LDs may also translocate into the lumen of bacteria-containing phagososmes. This has been shown for *M*. *leprae* which induces an accumulation of LDs within Schwann cells in close association with *M*. *leprae* containing phagosomes that are promptly recruited to the bacteria-containing phagosome in a PI3K signaling dependent process [18]. Similar phenomenon of LD-phagosome association has been also observed for *Chlamydia muridarum* [21] and *T*. *cruzi* [26].

Collectively our data demonstrate that besides our transcriptomic data, the confocal microscopy analysis shows that LDs formation within macrophages during *Leishmania* infection is not restricted to the parasite bearing cells suggesting the accumulation of LDs from a cellular origin. Our data also show the recruitment of LDs from the cytoplasm to the parasite-containing-parasitophorous vacuole and to the proximity of *Leishmania* parasites.

Although lipids play a key role in host defense, pathogens are clearly able to use lipids provided from the host for their proliferation [34]. Indeed, alteration of lipid metabolism during infection may allow the redistribution [35] or retention of nutrients such cholesterol in macrophages or other cells playing a key role in host defense [52]. On the other hand, different pathogens such *Chlamydia* [53], *Mycobacterium tuberculosis* [54] or *Toxoplasma gondii* [55] can use lipids to enter or survive within the cells. Altering lipid metabolism could help to control the outcome of infection. Indeed, it has been shown that elevated circulating cholesterol levels might be of benefit for *L*. *donovani* infections [56] while hypercholesterolemia increases the mortality of mice infected with *M*. *tuberculosis* [54].

A better characterization of macrophage/*Leishmania* LDs interactions mechanistic details, through approaches such as correlative large volume electron microscopy analysis of LDs interaction and dynamic combined with functional analysis [57], is critical for appropriate modulation of lipid metabolism in order to enhance the host’s normal defense mechanisms and allow the control of *Leishmania* infection.

## Supporting Information

S1 Fig Identification of activated genes in *L*. *major* infected macrophages in TG biosynthesis and breakdown pathway. (TIF)

S2 Fig Cholesterol accumulation diagram in *L*. *major* infected BMMs. (TIF)

S3 Fig Representative images of LD accumulation time course during *L*. *major* BMMs infection. BALB/c BMMs cells were infected by *Leishmania major* promastigotes at different time points. Cells were fixed by formaldehyde, stained by Bodipy493/503 for lipid droplet accumulation and Draq5 for parasite and cell nuclei and visualized by confocal microscopy. The results are representative for at least five independent experiments realized in duplicates.
(TIF)

S1 Table Canonical pathways identified by Inguenuity Pathway Analysis^™^ software as significantly altered (p < 0.05) in *L*. *major* infected BALB/c macrophages. (PDF)

S2 Table Gene expression of Lipid related genes in BALB/C Leishmania infected macrophages using qRT-PCR. A set of lipid related genes were tested by qRT-PCR. Changes in mRNA levels were calculated using the 2^−ΔΔCT^ method. The numbers presented in this table, for each time points are the average of three biological replicates.
(PDF)

S1 Video Three-dimensional representation of *L*.*major* infected macrophage using volume-rendering. LD localization in cell cytoplasm as well as in parasite cytoplasm is shown by Z-stack and 3D image rendering. Cells were subjected to anti-LPG for parasite outline (red), Bodipy for LD (green) and Draq5 for nuclei (bleu) staining.
(AVI)

S2 Video Time-lapse imaging of early BMMs parasite infection. LD accumulation is observed since 30mn of infection in cell cytoplasm and above intracellular parasites. BMMs were infected with transgenic Ds-Red or WT parasites for 30mn. Cells were stained with Bodipy for LD tracking. Confocal time-lapse imaging was performed at 37°C and images were taken every 15min for 18h.
(AVI)

S3 Video Time-lapse confocal images of late (24h) *L*. *major* BMMs infection. LDs are in constant movement in the cell cytoplasm and around parasites. BMMs were infected with transgenic Ds-Red or WT parasites for 24h. Extracellular parasites were taken off and cells were stained with Bodipy for LD tracking. Confocal time-lapse imaging was performed at 37°C and images were taken every 30min for 18h post infection. Scale bar = 12μm.
(AVI)

## References

[1] WHO. Leishmaniasis [Internet]. 2015 p. http://www.who.int/mediacentre/factsheets/fs375/en.

[2] Lecoeur H, Giraud E, Prévost MC, Milon G, Lang T. Reprogramming Neutral Lipid Metabolism in Mouse Dendritic Leucocytes Hosting Live Leishmania amazonensis Amastigotes. PLoS Negl Trop Dis. 2013;7 10.1371/journal.pntd.0002276 PMC3681733 23785538

[3] Rabhi I, Rabhi S, Ben-Othman R, Rasche A, Daskalaki A, Trentin B, et al Transcriptomic signature of Leishmania infected mice macrophages: a metabolic point of view. PLoS Negl Trop Dis. 2012;6: e1763 10.1371/journal.pntd.0001763 22928052 PMC3424254

[4] Osorio y Fortéa J, de La Llave E, Regnault B, Coppée J-Y, Milon G, Lang T, et al Transcriptional signatures of BALB/c mouse macrophages housing multiplying Leishmania amazonensis amastigotes. BMC Genomics. 2009;10: 119 10.1186/1471-2164-10-119 19302708 PMC2666765

[5] Bozza PT, Melo RCN, Bandeira-Melo C. Leukocyte lipid bodies regulation and function: contribution to allergy and host defense. Pharmacol Ther. 2007;113: 30–49. 10.1016/j.pharmthera.2006.06.006 16945418

[6] Melo RCN, D’Avila H, Wan H-C, Bozza PT, Dvorak AM, Weller PF. Lipid bodies in inflammatory cells: structure, function, and current imaging techniques. J Histochem Cytochem. 2011;59: 540–556. 21430261 10.1369/0022155411404073 PMC3201176

[7] Saka HA, Valdivia R. Emerging roles for lipid droplets in immunity and host-pathogen interactions. Annu Rev Cell Dev Biol. 2012;28: 411–37. 10.1146/annurev-cellbio-092910-153958 22578141

[8] Melo RCN, Dvorak AM. Lipid body-phagosome interaction in macrophages during infectious diseases: host defense or pathogen survival strategy? PLoS Pathog. 2012;8: e1002729 10.1371/journal.ppat.1002729 22792061 PMC3390411

[9] Barba G, Harper F, Harada T, Kohara M, Goulinet S, Matsuura Y, et al Hepatitis C virus core protein shows a cytoplasmic localization and associates to cellular lipid storage droplets. Proc Natl Acad Sci U S A. 1997;94: 1200–5. 9037030 10.1073/pnas.94.4.1200 PMC19768

[10] Harris C, Herker E, Farese R V, Ott M. Hepatitis C virus core protein decreases lipid droplet turnover: a mechanism for core-induced steatosis. J Biol Chem. 2011;286: 42615–25. 10.1074/jbc.M111.285148 21984835 PMC3234948

[11] Samsa MM, Mondotte JA, Iglesias NG, Assunção-Miranda I, Barbosa-Lima G, Da Poian AT, et al Dengue virus capsid protein usurps lipid droplets for viral particle formation. PLoS Pathog. 2009;5: e1000632 10.1371/journal.ppat.1000632 19851456 PMC2760139

[12] Peyron P, Vaubourgeix J, Poquet Y, Levillain F, Botanch C, Bardou F, et al Foamy macrophages from tuberculous patients’ granulomas constitute a nutrient-rich reservoir for M. tuberculosis persistence. PLoS Pathog. 2008;4: 1–14. 10.1371/journal.ppat.1000204 PMC2575403 19002241

[13] Daniel J, Maamar H, Deb C, Sirakova TD, Kolattukudy PE. Mycobacterium tuberculosis uses host triacylglycerol to accumulate lipid droplets and acquires a dormancy-like phenotype in lipid-loaded macrophages. PLoS Pathog. 2011;7 10.1371/journal.ppat.1002093 PMC3121879 21731490

[14] D’Avila H, Melo RCN, Parreira GG, Werneck-Barroso E, Castro-Faria-Neto HC, Bozza PT. Mycobacterium bovis bacillus Calmette-Guérin induces TLR2-mediated formation of lipid bodies: intracellular domains for eicosanoid synthesis in vivo. J Immunol. 2006;176: 3087–3097. 16493068 10.4049/jimmunol.176.5.3087

[15] D’Avila H, Roque NR, Cardoso RM, Castro-Faria-Neto HC, Melo RCN, Bozza PT. Neutrophils recruited to the site of Mycobacterium bovis BCG infection undergo apoptosis and modulate lipid body biogenesis and prostaglandin E2 production by macrophages. Cell Microbiol. 2008;10: 2589–2604. 10.1111/j.1462-5822.2008.01233.x 18771558

[16] Almeida PE, Silva AR, Maya-Monteiro CM, Töröcsik D, D’Avila H, Dezsö B, et al Mycobacterium bovis bacillus Calmette-Guérin infection induces TLR2-dependent peroxisome proliferator-activated receptor gamma expression and activation: functions in inflammation, lipid metabolism, and pathogenesis. J Immunol. 2009;183: 1337–1345. 10.4049/jimmunol.0900365 19561094

[17] Mattos K a, D’Avila H, Rodrigues LS, Oliveira VGC, Sarno EN, Atella GC, et al Lipid droplet formation in leprosy: Toll-like receptor-regulated organelles involved in eicosanoid formation and Mycobacterium leprae pathogenesis. J Leukoc Biol. 2010;87: 371–384. 10.1189/jlb.0609433 19952355

[18] Mattos KA, Lara FA, Oliveira VGC, Rodrigues LS, D’Avila H, Melo RCN, et al Modulation of lipid droplets by Mycobacterium leprae in Schwann cells: a putative mechanism for host lipid acquisition and bacterial survival in phagosomes. Cell Microbiol. 2011;13: 259–273. 10.1111/j.1462-5822.2010.01533.x 20955239

[19] de Mattos KA, Sarno EN, Pessolani MCV, Bozza PT. Deciphering the contribution of lipid droplets in leprosy: multifunctional organelles with roles in Mycobacterium leprae pathogenesis. Mem Inst Oswaldo Cruz. 2012;107 Suppl: 156–66. Available: http://www.ncbi.nlm.nih.gov/pubmed/23283467 23283467 10.1590/s0074-02762012000900023

[20] Cao F, Castrillo A, Tontonoz P, Re F, Byrne GI. Chlamydia pneumoniae-induced macrophage foam cell formation is mediated by toll-like receptor 2. Infect Immun. 2007;75: 753–759. 10.1128/IAI.01386-06 17145941 PMC1828523

[21] Rank RG, Whittimore J, Bowlin AK, Wyrick PB. In vivo ultrastructural analysis of the intimate relationship between polymorphonuclear leukocytes and the chlamydial developmental cycle. Infect Immun. 2011;79: 3291–301. 10.1128/IAI.00200-11 21576327 PMC3147583

[22] Kumar Y, Cocchiaro J, Valdivia RH. The obligate intracellular pathogen Chlamydia trachomatis targets host lipid droplets. Curr Biol. 2006;16: 1646–51. 10.1016/j.cub.2006.06.060 16920627

[23] Nawabi P, Catron DM, Haldar K. Esterification of cholesterol by a type III secretion effector during intracellular Salmonella infection. Mol Microbiol. 2008;68: 173–85. 10.1111/j.1365-2958.2008.06142.x 18333886

[24] Khatchadourian A, Bourque SD, Richard VR, Titorenko VI, Maysinger D. Dynamics and regulation of lipid droplet formation in lipopolysaccharide (LPS)-stimulated microglia. Biochim Biophys Acta—Mol Cell Biol Lipids. Elsevier B.V.; 2012;1821: 607–617. 10.1016/j.bbalip.2012.01.007 22289388

[25] Melo RCN, D’Avila H, Fabrino DL, Almeida PE, Bozza PT. Macrophage lipid body induction by Chagas disease in vivo: putative intracellular domains for eicosanoid formation during infection. Tissue Cell. 2003;35: 59–67. 10.1016/S0040-8166(02)00105-2 12589730

[26] D’Avila H, Freire-de-Lima CG, Roque NR, Teixeira L, Barja-Fidalgo C, Silva AR, et al Host cell lipid bodies triggered by Trypanosoma cruzi infection and enhanced by the uptake of apoptotic cells are associated with prostaglandin E2 generation and increased parasite growth. J Infect Dis. 2011;204: 951–961. 10.1093/infdis/jir432 21849292

[27] Rodríguez-Acosta A, Finol HJ, Pulido-Méndez M, Márquez A, Andrade G, González N, et al Liver ultrastructural pathology in mice infected with Plasmodium berghei. J Submicrosc Cytol Pathol. 1998;30: 299–307. 9648294

[28] Pulido-Méndez M, Finol HJ, Girón ME, Aguilar I. Ultrastructural pathological changes in mice kidney caused by Plasmodium berghei infection. J Submicrosc Cytol Pathol. 38: 143–8. 17784642

[29] Charron AJ, Sibley LD. Host cells: mobilizable lipid resources for the intracellular parasite Toxoplasma gondii. J Cell Sci. 2002;115: 3049–3059. 12118061 10.1242/jcs.115.15.3049

[30] Pinheiro RO, Nunes MP, Pinheiro CS, D’Avila H, Bozza PT, Takiya CM, et al Induction of autophagy correlates with increased parasite load of Leishmania amazonensis in BALB/c but not C57BL/6 macrophages. Microbes Infect. 2009;11: 181–190. 10.1016/j.micinf.2008.11.006 19070676

[31] Martin S, Parton RG. Caveolin, cholesterol, and lipid bodies. Semin Cell Dev Biol. 2005;16: 163–174. 10.1016/j.semcdb.2005.01.007 15797827

[32] Martin S, Parton RG. Lipid droplets: a unified view of a dynamic organelle. Nat Rev Mol Cell Biol. 2006;7: 373–378. 10.1038/nrm1912 16550215

[33] Farese R V. NIH Public Access. Cell. 2011;139: 855–860.

[34] Herker E, Ott M. Emerging role of lipid droplets in host/pathogen interactions. J Biol Chem. 2012;287: 2280–7. 10.1074/jbc.R111.300202 22090026 PMC3268388

[35] Khovidhunkit W, Kim M-S, Memon RA, Shigenaga JK, Moser AH, Feingold KR, et al Effects of infection and inflammation on lipid and lipoprotein metabolism: mechanisms and consequences to the host. J Lipid Res. 2004;45: 1169–1196. 15102878 10.1194/jlr.R300019-JLR200

[36] Feingold KR, Grunfeld C. Lipids: a key player in the battle between the host and microorganisms. J Lipid Res. 2012;53: 2487–9. 10.1194/jlr.E033407 23075464 PMC3494250

[37] De Cicco NNT, Pereira MG, Corrêa JR, Andrade-Neto V V., Saraiva FB, Chagas-Lima AC, et al LDL uptake by Leishmania amazonensis: Involvement of membrane lipid microdomains. Exp Parasitol. Elsevier Inc.; 2012;130: 330–340. 10.1016/j.exppara.2012.02.014 22381219

[38] Araújo-Santos T, Rodríguez NE, de Moura Pontes S, Dixt UG, Abánades DR, Bozza PT, et al Role of prostaglandin F2α production in lipid bodies from Leishmania infantum chagasi: insights on virulence. J Infect Dis. 2014;210: 1951–1961. 10.1093/infdis/jiu299 24850789 PMC6281352

[39] Polando R, Dixit UG, Carter CR, Jones B, Whitcomb JP, Ballhorn W, et al The roles of complement receptor 3 and Fcγ receptors during Leishmania phagosome maturation. J Leukoc Biol. 2013;93: 921–32. 10.1189/jlb.0212086 23543768 PMC3656333

[40] Kellner-Weibel G, McHendry-Rinde B, Haynes MP, Adelman S. Evidence that newly synthesized esterified cholesterol is deposited in existing cytoplasmic lipid inclusions. J Lipid Res. 2001;42: 768–777. 11352984

[41] Harris CA, Haas JT, Streeper RS, Stone SJ, Kumari M, Yang K, et al DGAT enzymes are required for triacylglycerol synthesis and lipid droplets in adipocytes. J Lipid Res. 2011;52: 657–67. 10.1194/jlr.M013003 21317108 PMC3284159

[42] Xu N, Zhang SO, Cole RA, McKinney SA, Guo F, Haas JT, et al The FATP1-DGAT2 complex facilitates lipid droplet expansion at the ER-lipid droplet interface. J Cell Biol. 2012;198: 895–911. 10.1083/jcb.201201139 22927462 PMC3432760

[43] Bozza PT, Magalhães KG, Weller PF. Leukocyte lipid bodies—Biogenesis and functions in inflammation. Biochim Biophys Acta—Mol Cell Biol Lipids. Elsevier B.V.; 2009;1791: 540–551. 10.1016/j.bbalip.2009.01.005 PMC2693476 19416659

[44] França-Costa J, Van Weyenbergh J, Boaventura VS, Luz NF, Malta-Santos H, Oliveira MCS, et al Arginase I, polyamine, and prostaglandin E2 pathways suppress the inflammatory response and contribute to diffuse cutaneous leishmaniasis. J Infect Dis. 2015;211: 426–35. 10.1093/infdis/jiu455 25124926

[45] Passero LFD, Laurenti MD, Tomokane TY, Corbett CEP, Toyama MH. The effect of phospholipase A2 from Crotalus durissus collilineatus on Leishmania (Leishmania) amazonensis infection. Parasitol Res. 2008;102: 1025–33. 10.1007/s00436-007-0871-6 18180953

[46] Das P, De T, Chakraborti T. Leishmania donovani secretory serine protease alters macrophage inflammatory response via COX-2 mediated PGE-2 production. Indian J Biochem Biophys. 2014;51: 542–51. Available: http://www.ncbi.nlm.nih.gov/pubmed/25823228 25823228

[47] Matte C, Maion G, Mourad W, Olivier M. Leishmania donovani-induced macrophages cyclooxygenase-2 and prostaglandin E2 synthesis. Parasite Immunol. 2001;23: 177–184. 10.1046/j.1365-3024.2001.00372.x 11298294

[48] Jaramillo M, Gomez MA, Larsson O, Shio MT, Topisirovic I, Contreras I, et al Leishmania repression of host translation through mTOR cleavage is required for parasite survival and infection. Cell Host Microbe. 2011;9: 331–41. 10.1016/j.chom.2011.03.008 21501832

[49] Vinet AF, Fukuda M, Turco SJ, Descoteaux A. The Leishmania donovani lipophosphoglycan excludes the vesicular proton-ATPase from phagosomes by impairing the recruitment of synaptotagmin V. PLoS Pathog. 2009;5: e1000628 10.1371/journal.ppat.1000628 19834555 PMC2757729

[50] Matheoud D, Moradin N, Bellemare-Pelletier A, Shio MT, Hong WJ, Olivier M, et al Leishmania evades host immunity by inhibiting antigen cross-presentation through direct cleavage of the SNARE VAMP8. Cell Host Microbe. 2013;14: 15–25. 10.1016/j.chom.2013.06.003 23870310

[51] Melo RCN, Fabrino DL, Dias FF, Parreira GG. Lipid bodies: Structural markers of inflammatory macrophages in innate immunity. Inflamm Res. 2006;55: 342–8. 10.1007/s00011-006-5205-0 16977381

[52] Feingold KR, Grunfeld C. The acute phase response inhibits reverse cholesterol transport. J Lipid Res. 2010;51: 682–4. 10.1194/jlr.E005454 20071695 PMC2842157

[53] Bashmakov YK, Zigangirova NA, Pashko YP, Kapotina LN, Petyaev IM. Chlamydia trachomatis growth inhibition and restoration of LDL-receptor level in HepG2 cells treated with mevastatin. Comp Hepatol. 2010;9: 3 10.1186/1476-5926-9-3 20181044 PMC2835644

[54] Martens GW, Arikan MC, Lee J, Ren F, Vallerskog T, Kornfeld H. Hypercholesterolemia impairs immunity to tuberculosis. Infect Immun. 2008;76: 3464–72. 10.1128/IAI.00037-08 18505807 PMC2493195

[55] Portugal LR, Fernandes LR, Pietra Pedroso VS, Santiago HC, Gazzinelli RT, Alvarez-Leite JI. Influence of low-density lipoprotein (LDL) receptor on lipid composition, inflammation and parasitism during Toxoplasma gondii infection. Microbes Infect. 2008;10: 276–284. 10.1016/j.micinf.2007.12.001 18316222

[56] Ghosh J, Das S, Guha R, Ghosh D, Naskar K, Das A, et al Hyperlipidemia offers protection against Leishmania donovani infection: role of membrane cholesterol. J Lipid Res. 2012;53: 2560–72. 10.1194/jlr.M026914 23060454 PMC3494257

[57] Mellouk N, Weiner A, Aulner N, Schmitt C, Elbaum M, Shorte SL, et al Shigella Subverts the Host Recycling Compartment to Rupture Its Vacuole. Cell Host Microbe. Elsevier Inc.; 2014;16: 517–530. 10.1016/j.chom.2014.09.005 25299335

