# Supplementary figures and images for: Lipid Droplet Formation, Their Localization and Dynamics during Leishmania major Macrophage Infection
Source: PLoS One. 2016 Feb 12;11(2):e0148640. doi: 10.1371/journal.pone.0148640 (PMC4752496; doi:10.1371/journal.pone.0148640)

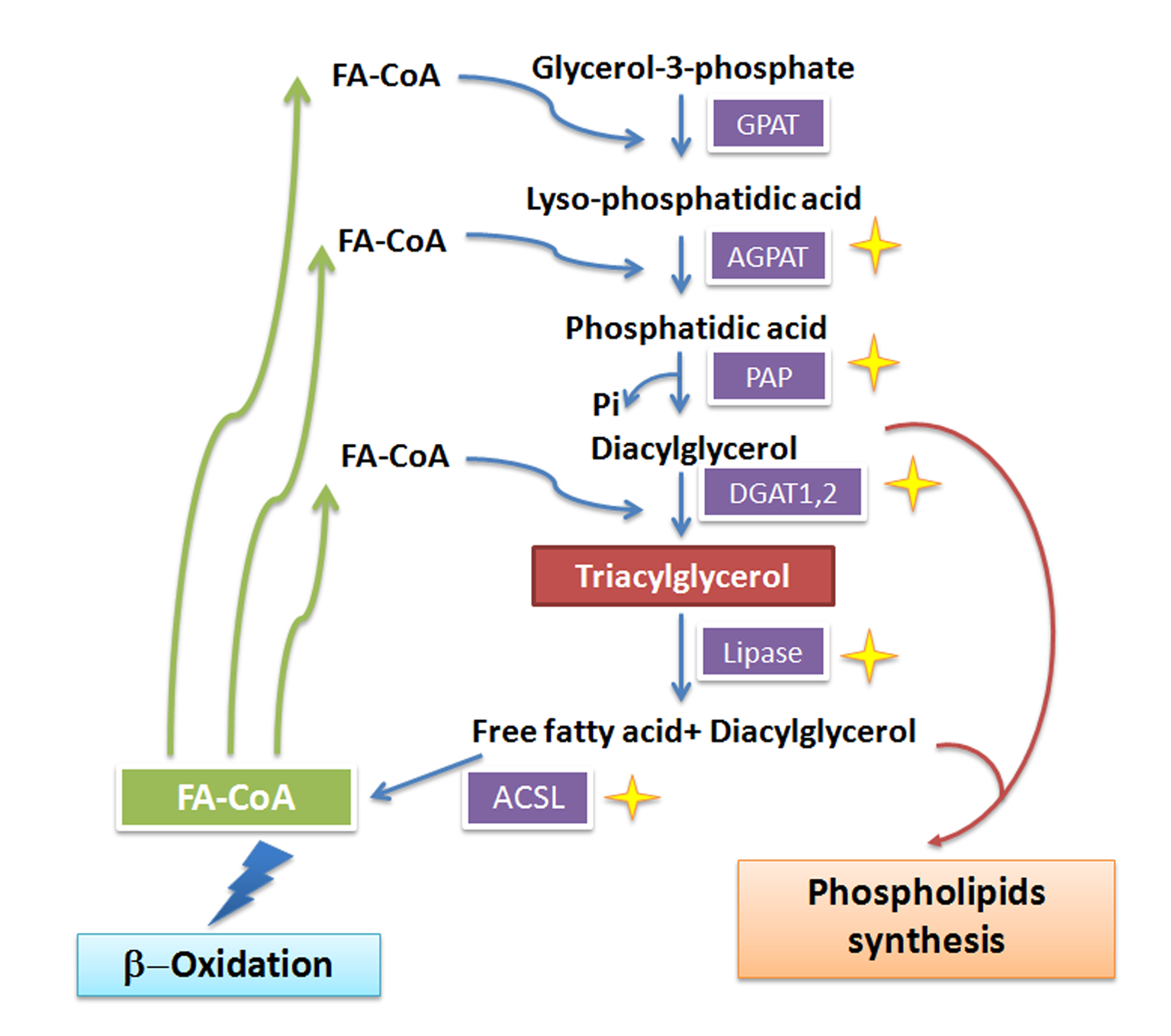

Supplement: S1 Fig — (TIF) [file pone.0148640.s001.tif]

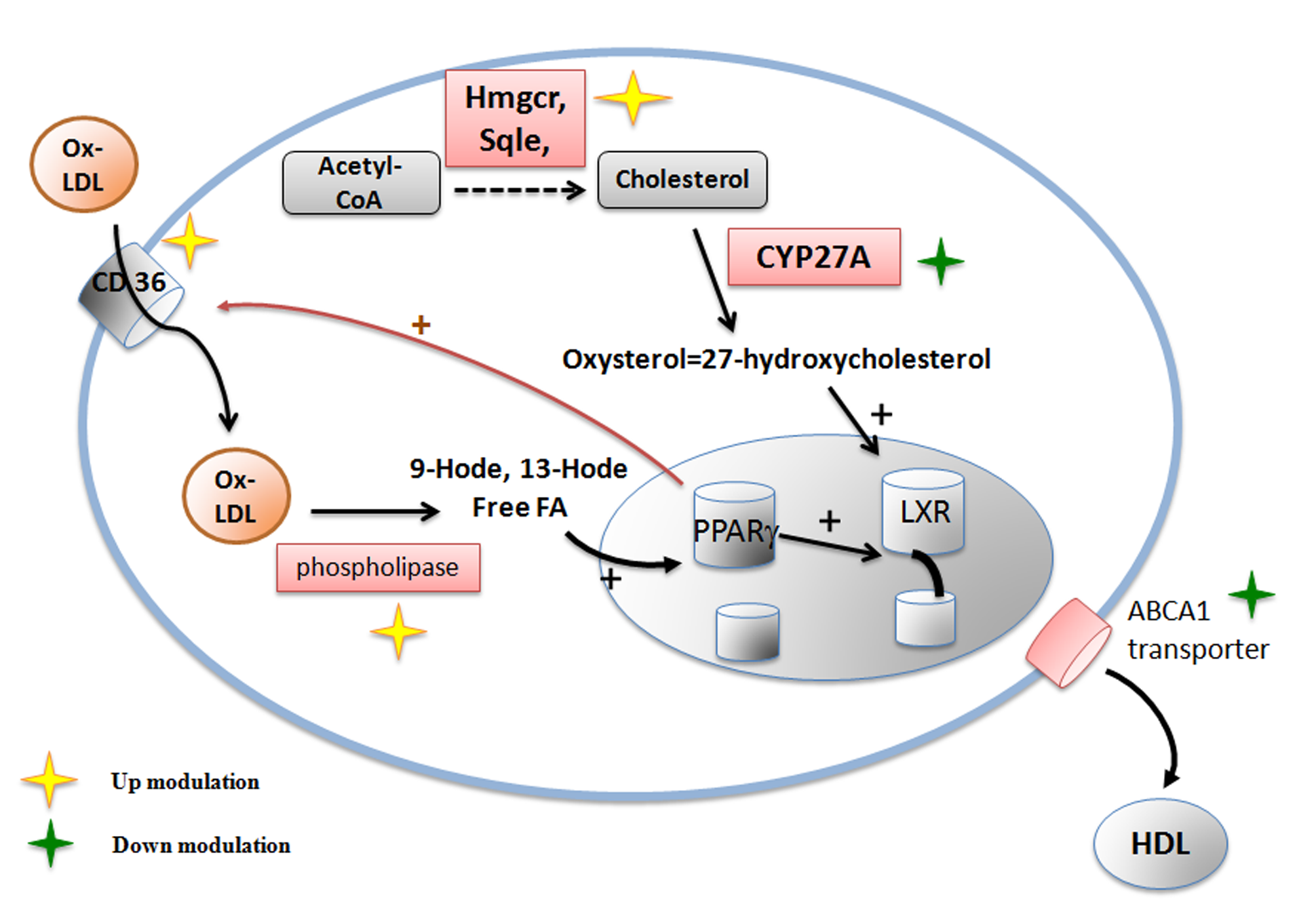

Supplement: S2 Fig — (TIF) [file pone.0148640.s002.tif]

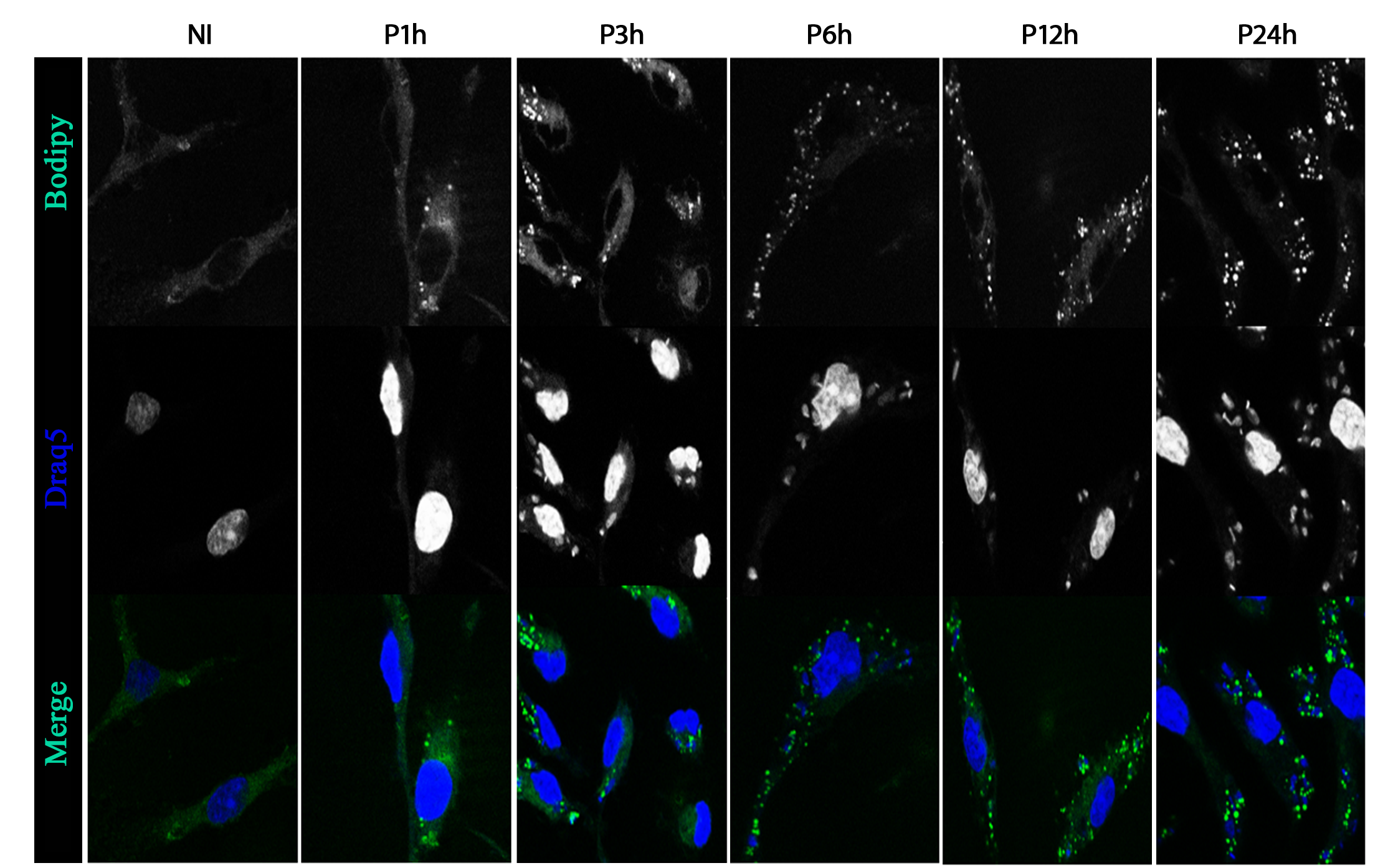

Supplement: S3 Fig — BALB/c BMMs cells were infected by Leishmania major promastigotes at different time points. Cells were fixed by formaldehyde, stained by Bodipy493/503 for lipid droplet accumulation and Draq5 for parasite and cell nuclei and visualized by confocal microscopy. The results are representative for at least five independent experiments realized in duplicates. (TIF) [file pone.0148640.s003.tif]
