# Supplementary material for: Lipid Droplet Formation, Their Localization and Dynamics during Leishmania major Macrophage Infection
Source: PLoS One. 2016 Feb 12;11(2):e0148640. doi: 10.1371/journal.pone.0148640 (PMC4752496; doi:10.1371/journal.pone.0148640)
Supplement: S1 Table — (PDF) [file pone.0148640.s004.pdf]

© 2000-2009 Ingenuity Systems, Inc. All rights reserved.

Ingenuity Canonical Pathways	AnalysisName	-log(p-value)	Ratio	Molecules
Glycerolipid Metabolism	3h	7,4E00	3,85E-02	AGPAT5, GLA, ALDH3A2, PPAP2B, PNPLA2, PTGR1
Glycerolipid Metabolism	6h	7,4E00	3,85E-02	AGPAT5, GLA, ALDH3A2, PPAP2B, PNPLA2, PTGR1
Glycerolipid Metabolism	12h	7,4E00	3,85E-02	AGPAT5, GLA, ALDH3A2, PPAP2B, PNPLA2, PTGR1
Glycerolipid Metabolism	24h	7,4E00	3,85E-02	AGPAT5, GLA, ALDH3A2, PPAP2B, PNPLA2, PTGR1
Eicosanoid Signaling	3h	6,63E00	5,95E-02	PLA2G4A, PTGES, PTGS1, PTGS2, PTGER4
Eicosanoid Signaling	6h	6,63E00	5,95E-02	PLA2G4A, PTGES, PTGS1, PTGS2, PTGER4
Eicosanoid Signaling	12h	6,63E00	5,95E-02	PLA2G4A, PTGES, PTGS1, PTGS2, PTGER4
Eicosanoid Signaling	24h	6,63E00	5,95E-02	PLA2G4A, PTGES, PTGS1, PTGS2, PTGER4
Arachidonic Acid Metabolism	3h	5,22E00	2,2E-02	PLA2G4A, PTGES, PTGES3 (includes EG:10728), PTGS1, PTGS2
Arachidonic Acid Metabolism	6h	5,22E00	2,2E-02	PLA2G4A, PTGES, PTGES3 (includes EG:10728), PTGS1, PTGS2
Arachidonic Acid Metabolism	12h	5,22E00	2,2E-02	PLA2G4A, PTGES, PTGES3 (includes EG:10728), PTGS1, PTGS2
Arachidonic Acid Metabolism	24h	5,22E00	2,2E-02	PLA2G4A, PTGES, PTGES3 (includes EG:10728), PTGS1, PTGS2
LPS/IL-1 Mediated Inhibition of RXR Fun	3h	4,53E00	2,33E-02	ALDH3A2, FABP4, ABCC3, ACSL1, ABCA1
LPS/IL-1 Mediated Inhibition of RXR Fun	6h	4,53E00	2,33E-02	ALDH3A2, FABP4, ABCC3, ACSL1, ABCA1
LPS/IL-1 Mediated Inhibition of RXR Fun	12h	4,53E00	2,33E-02	ALDH3A2, FABP4, ABCC3, ACSL1, ABCA1
LPS/IL-1 Mediated Inhibition of RXR Fun	24h	4,53E00	2,33E-02	ALDH3A2, FABP4, ABCC3, ACSL1, ABCA1
Bile Acid Biosynthesis	3h	4,04E00	3E-02	CYP27A1, ALDH3A2, PTGR1
Bile Acid Biosynthesis	6h	4,04E00	3E-02	CYP27A1, ALDH3A2, PTGR1
Bile Acid Biosynthesis	12h	4,04E00	3E-02	CYP27A1, ALDH3A2, PTGR1
Bile Acid Biosynthesis	24h	4,04E00	3E-02	CYP27A1, ALDH3A2, PTGR1
Glycerophospholipid Metabolism	3h	3,86E00	2,07E-02	PLA2G4A, AGPAT5, LPCAT2, PPAP2B
Glycerophospholipid Metabolism	6h	3,86E00	2,07E-02	PLA2G4A, AGPAT5, LPCAT2, PPAP2B
Glycerophospholipid Metabolism	12h	3,86E00	2,07E-02	PLA2G4A, AGPAT5, LPCAT2, PPAP2B
Glycerophospholipid Metabolism	3h	3,86E00	2,07E-02	PLA2G4A, AGPAT5, LPCAT2, PPAP2B
Sphingolipid Metabolism	6h	3,24E00	2,68E-02	GLA, PPAP2B, SGPP1
Sphingolipid Metabolism	12h	3,24E00	2,68E-02	GLA, PPAP2B, SGPP1
Sphingolipid Metabolism	24h	3,24E00	2,68E-02	GLA, PPAP2B, SGPP1
Sphingolipid Metabolism	3h	3,24E00	2,68E-02	GLA, PPAP2B, SGPP1
Glycolysis/Gluconeogenesis	6h	3,08E00	2,11E-02	ALDH3A2, PTGR1, ACSL1
Glycolysis/Gluconeogenesis	12h	3,08E00	2,11E-02	ALDH3A2, PTGR1, ACSL1
Glycolysis/Gluconeogenesis	3h	3,08E00	2,11E-02	ALDH3A2, PTGR1, ACSL1
Glycolysis/Gluconeogenesis	6h	3,08E00	2,11E-02	ALDH3A2, PTGR1, ACSL1
Xenobiotic Metabolism Signaling	12h	2,82E00	1,37E-02	PNPLA7, PTGES3 (includes EG:10728), ALDH3A2, ABCC3
Xenobiotic Metabolism Signaling	3h	2,82E00	1,37E-02	PNPLA7, PTGES3 (includes EG:10728), ALDH3A2, ABCC3
Xenobiotic Metabolism Signaling	6h	2,82E00	1,37E-02	PNPLA7, PTGES3 (includes EG:10728), ALDH3A2, ABCC3
Xenobiotic Metabolism Signaling	12h	2,82E00	1,37E-02	PNPLA7, PTGES3 (includes EG:10728), ALDH3A2, ABCC3
Fatty Acid Metabolism	3h	2,8E00	1,56E-02	ALDH3A2, PTGR1, ACSL1
Fatty Acid Metabolism	6h	2,8E00	1,56E-02	ALDH3A2, PTGR1, ACSL1
Fatty Acid Metabolism	12h	2,8E00	1,56E-02	ALDH3A2, PTGR1, ACSL1
Fatty Acid Metabolism	3h	2,8E00	1,56E-02	ALDH3A2, PTGR1, ACSL1
Hepatic Cholestasis	6h	2,64E00	1,8E-02	CYP27A1, ABCC1, ABCC3
Hepatic Cholestasis	12h	2,64E00	1,8E-02	CYP27A1, ABCC1, ABCC3
Hepatic Cholestasis	3h	2,64E00	1,8E-02	CYP27A1, ABCC1, ABCC3
Hepatic Cholestasis	6h	2,64E00	1,8E-02	CYP27A1, ABCC1, ABCC3
MIF Regulation of Innate Immunity	12h	2,57E00	4,35E-02	PLA2G4A, PTGS2
MIF Regulation of Innate Immunity	3h	2,57E00	4,35E-02	PLA2G4A, PTGS2
MIF Regulation of Innate Immunity	6h	2,57E00	4,35E-02	PLA2G4A, PTGS2
MIF Regulation of Innate Immunity	12h	2,57E00	4,35E-02	PLA2G4A, PTGS2
Endothelin-1 Signaling	3h	2,3E00	1,61E-02	PLA2G4A, PTGS1, PTGS2
Endothelin-1 Signaling	6h	2,3E00	1,61E-02	PLA2G4A, PTGS1, PTGS2
Endothelin-1 Signaling	12h	2,3E00	1,61E-02	PLA2G4A, PTGS1, PTGS2
Endothelin-1 Signaling	3h	2,3E00	1,61E-02	PLA2G4A, PTGS1, PTGS2
PXR/RXR Activation	6h	2,17E00	2,2E-02	ALDH3A2, ABCC3
PXR/RXR Activation	12h	2,17E00	2,2E-02	ALDH3A2, ABCC3
PXR/RXR Activation	3h	2,17E00	2,2E-02	ALDH3A2, ABCC3
PXR/RXR Activation	6h	2,17E00	2,2E-02	ALDH3A2, ABCC3
LXR/RXR Activation	3h	2,09E00	2,33E-02	CD36, ABCA1
LXR/RXR Activation	6h	2,09E00	2,33E-02	CD36, ABCA1
LXR/RXR Activation	12h	2,09E00	2,33E-02	CD36, ABCA1
LXR/RXR Activation	3h	2,09E00	2,33E-02	CD36, ABCA1
Propanoate Metabolism	6h	2,09E00	1,54E-02	ALDH3A2, ACSL1
Propanoate Metabolism	12h	2,09E00	1,54E-02	ALDH3A2, ACSL1
Propanoate Metabolism	24h	2,09E00	1,54E-02	ALDH3A2, ACSL1
Propanoate Metabolism	3h	2,09E00	1,54E-02	ALDH3A2, ACSL1
Pyruvate Metabolism	6h	1,97E00	1,34E-02	ALDH3A2, ACSL1
Pyruvate Metabolism	12h	1,97E00	1,34E-02	ALDH3A2, ACSL1
Pyruvate Metabolism	24h	1,97E00	1,34E-02	ALDH3A2, ACSL1
Pyruvate Metabolism	3h	1,97E00	1,34E-02	ALDH3A2, ACSL1
Linoleic Acid Metabolism	6h	1,91E00	1,61E-02	PLA2G4A, FADS1
Linoleic Acid Metabolism	12h	1,91E00	1,61E-02	PLA2G4A, FADS1
Linoleic Acid Metabolism	3h	1,91E00	1,61E-02	PLA2G4A, FADS1
Linoleic Acid Metabolism	3h	1,91E00	1,61E-02	PLA2G4A, FADS1
Inositol Metabolism	6h	1,87E00	2,04E-02	DEGS1, FADS1
Inositol Metabolism	12h	1,87E00	2,04E-02	DEGS1, FADS1
Inositol Metabolism	3h	1,87E00	2,04E-02	DEGS1, FADS1
Inositol Metabolism	6h	1,87E00	2,04E-02	DEGS1, FADS1
Phospholipid Degradation	12h	1,86E00	1,89E-02	PLA2G4A, PPAP2B
Phospholipid Degradation	3h	1,86E00	1,89E-02	PLA2G4A, PPAP2B
Phospholipid Degradation	6h	1,86E00	1,89E-02	PLA2G4A, PPAP2B
Phospholipid Degradation	12h	1,86E00	1,89E-02	PLA2G4A, PPAP2B
