# Supplementary material for: Lipid Droplet Formation, Their Localization and Dynamics during Leishmania major Macrophage Infection
Source: PLoS One. 2016 Feb 12;11(2):e0148640. doi: 10.1371/journal.pone.0148640 (PMC4752496; doi:10.1371/journal.pone.0148640)
Supplement: S2 Table — A set of lipid related genes were tested by qRT-PCR. Changes in mRNA levels were calculated using the 2−ΔΔCT method. The numbers presented in this table, for each time points are the average of three biological replicates. (PDF) [file pone.0148640.s005.pdf]

Gene expression of Lipid related genes in BALB/c Leishmania infected macrophages using qRT-PCR

The numbers presented are annotated as 2 -DD Ct=2power-DDCt
2 -DD Ct=2power-DDCt
DDCt=DCt -DCt NI (t: point of the infection)
DeltaCt=Ct gene of interest-Ct of the reference gene.
Please note that 4 reference genes were used for the qPCR normalization.

NI=Non infected BALB/c macrophages
P: BALB/c macrophages infected with live parasite
KP: BALB/c macrophage infected with killed parasites

MGI Symbol		P 1hBALB/c	P 3hBALB/c	P 6hBALB/c	P 12hBALB/c	P 24hBALB/c	KP 1hBALB/c	KP 3hBALB/c	KP 6hBALB/c	KP 12hBALB/c	KP 24hBALB/c
Hmgcr	Chholesterol pathway	1.357089074	1.555692223	1.4248981	0.832287188	0.867140396	1.259738759	2.225907025	1.34105222	1.304835104	0.710381254
Sqle		1.329481544	2.522871396	3.117941262	1.453057372	1.4464193	1.37420406	1.869207417	1.452292534	1.247097418	1.350587475
Cyp27a1		0.846888141	0.660293035	0.171129553	0.446472792	0.462208811	0.789867007	0.845118421	0.318366219	0.867173705	0.74717599
Abca1		0.851528713	0.877210509	0.673473811	0.834468791	0.757446467	0.974690609	1.161055283	0.810096352	1.003053924	0.76790447
Scd2		0.939264464	1.008035653	1.779681616	1.867332924	2.871517459	0.864010051	1.035138456	1.228197439	1.137498453	2.767103614
Cd36		0.84873167	1.435616092	2.286641847	1.96686781	2.485393297	0.845080834	2.401570787	4.476978583	5.99473439	3.182110093
Cav1		0.967616371	1.01872289	2.162055788	1.338900112	0.674514253	1.310720052	0.618459059	0.567829123	0.571662608	0.519513118
Lrp12		0.934865301	1.705248114	1.424504735	0.825000367	1.093620734	1.017623987	2.223470607	1.875792722	1.136302932	0.871131297
Acsl1		1.12963661	2.153497527	3.623177327	1.438837169	1.39693591	1.394917292	2.249771271	2.100404342	1.342667893	0.950430968
Fabp4		1.21260142	2.204021555	1.99529042	1.21761525	1.21945301	1.407654148	3.579467548	3.144950006	1.029783339	0.78044223
Acsf2		0.843274133	0.615084017	0.47837677	0.707271102	0.579083659	0.732462139	0.600489082	0.661302986	0.830951339	0.573307553
Lpl		0.6334578	0.87081309	0.92580617	1.684563719	2.948509481	0.684421151	1.988972506	4.390830217	6.086456315	3.566450005
Ppap2b	Triglyceride pathway	1.161150519	4.356873825	7.421517281	2.221543668	2.341151559	1.311085405	2.684088207	5.54353341	3.65462805	1.259611063
Dgat2		0.764091376	2.699664261	1.270035435	0.826937386	0.803812453	0.975529846	2.728765336	1.014474135	1.495603238	0.73544299
Agpat9		1.000475398	3.486386561	3.651301786	2.262230229	1.316751894	0.862586245	1.618455284	1.047598704	0.949280481	1.822762125
Agpat5		0.902617768	2.198086763	1.832792868	0.963083862	1.028357933	1.060633521	1.510896672	1.176958744	0.908009418	1.000024549
